# Bacterial alarmone (p)ppGpp mediates the pathogenicity of *Clavibacter michiganensis* via a dual mechanism that affects both enzyme production and the Tat secretion system

**DOI:** 10.1128/msystems.00135-25

**Published:** 2025-08-04

**Authors:** Xiaoli Xu, Kaihong Bai, Jia Shi, Chengxuan Yu, Shuang Song, Na Jiang, Jianqiang Li, Laixin Luo

**Affiliations:** 1 Department of Plant Pathology, College of Plant Protection/Beijing Key Laboratory of Seed Disease Testing and Control, China Agricultural University 630134, Beijing, China; 2 School of Life Sciences, Zhengzhou University 12636 , Zhengzhou, Henan, China; International Rice Research Institute, Manila, Philippines

**Keywords:** (p)ppGpp, *Clavibacter michiganensis*, pathogenicity factors, Twin-arginine translocation system, xylanase secretion

## Abstract

The gram-positive bacterium *Clavibacter michiganensis* (Cm) causes bacterial canker of tomato. The infection process of Cm is of considerable interest, and the role of the alarmone guanosine tetraphosphate or pentaphosphate [(p)ppGpp], a global regulator, has been strongly implicated in bacterial survival and pathogenicity. Transcriptome analysis comparing a (p)ppGpp-deficient strain (Δ*rel*) to the wild-type strain demonstrated that (p)ppGpp down-regulates the expression of many genes encoding ribosomal components and ABC transporter proteins, while up-regulating genes associated with amino acid metabolism, biofilm synthesis, and the production and secretion of cell-wall degrading enzymes. Biochemical assays showed reduced biofilm synthesis and extracellular activity of cell-wall degrading enzymes such as amylase and xylanase. Moreover, the results of microscale thermophoresis and electrophoretic mobility shift assays indicated that (p)ppGpp interacts with the transcription factor Vatr1 to directly suppress expression of the xylanase gene *xysB*. Meanwhile, amidase reporter assays revealed that xylanase secretion depends on the Tat secretion system, which was significantly inhibited in Δ*rel*, leading to intracellular accumulation of the xylanase protein. Taken together, these results indicate that (p)ppGpp plays a complex role in the pathogenicity of *C. michiganensis*, mediating, among other things, not only the production of cell-wall degrading enzymes but also their transport and secretion.

**IMPORTANCE:**

This study reveals the pivotal role of the bacterial alarmone guanosine tetraphosphate and guanosine pentaphosphate in the pathogenicity of *Clavibacter michiganensis*, a significant plant pathogen. Through the identification of its dual mechanisms in regulating enzyme production and the Tat secretion system, we uncover key insights into bacterial virulence strategies. Our findings not only advance the understanding of bacterial stress response systems but also offer new opportunities for developing targeted interventions to combat plant bacterial diseases, ultimately contributing to agricultural sustainability and food security.

## INTRODUCTION

The gram-positive bacterium *Clavibacter michiganensis* (Cm) is the causative agent of tomato bacterial canker, a disease that greatly impacts global tomato production (1, 2). The primary infection can result from numerous sources, including contaminated seeds, tomato debris, symptomless but infected tomato transplants, and alternative hosts (3, 4). However, once infected, Cm quickly colonizes the xylem of its tomato host to produce a systemic infection that results in symptoms of leaf wilt and necrosis, as well as the formation of cankers on the stem and petioles. Severe infections can lead to plant death, but even localized infection can cause bird’s-eye lesions on tomato fruit that reduce the quality and value of the crop (5, 6). Furthermore, Cm does not just impact crop production *per se*, but also presents a major threat to the seed and planting stock industry, and consequently, Cm is internationally recognized as a quarantine pest that requires strict import and export regulations, since there are few disease interventions such as resistant varieties or anti-bacterial compounds, and therefore, control relies primarily on avoiding infected material.

Many aspects of the Cm life cycle have been studied in detail, and it is well understood that after initial infection, the pathogen quickly multiplies within the xylem to levels as high as 10^9-10^ colony-forming units per gram of fresh tissue (7, 8). The vascular tissues of an infected plant can be entirely filled with bacterial cells, leading to vascular collapse and the wilting of leaves as the flow of water is disrupted (2, 9). Unlike gram-negative plant pathogens, which utilize type II or type III secretion systems (T2SS and T3SS) to secrete a large set of plant cell-wall degrading enzymes (CWDEs) and type III effectors (T3Es) into the plant tissue, Cm, which lacks a T3SS, relies on the Sec or Twin-arginine protein translocation pathway to release its arsenal of CWDEs (1, 9–13). Furthermore, it is known that the reference Cm strain, NCPPB382, contains a 129 kb pathogenicity island in its genome called *chp/tomA* pathogenicity island (PAI), as well as two circular conjugative plasmids, pCM1 (~27.5 kb) and pCM2 (~70 kb), which are required for full pathogenicity in tomato plants (14). The *chp* region itself is approximately 79 kb long and mainly encodes hydrolytic enzymes such as serine proteases, glycoside hydrolases (GHs), and pectate lyases, while the *tomA* sub-region spans approximately 50 kb and has a high gene density that mainly encodes proteins associated with the recognition and uptake of carbohydrate (14). Investigation of the CMM30-18 and Cmm27 deletion mutants derived from NCPPB382, which lack the *chp/tomA* PAI, revealed that they are non-virulent and unable to colonize the tomato host effectively (14–16). Meanwhile, other studies have found that most Cm strains contain two additional plasmids, with pCM1 encoding the *celA* cellulase gene, which is considered a key pathogenic gene, while pCM2 contains another crucial virulence gene *pat-1* that encodes a serine protease. Although deletion of either plasmid greatly reduces the pathogenicity of Cm, neither is absolutely required to form an infection (1, 16). In addition to these genetic elements that are directly linked to pathogenicity, research has shown that the Cm genome contains many genes encoding transcription factors, including Vatr1 and Vatr2, which seem to play important regulatory roles during the Cm infection (17).

The ability of pathogenic bacteria to counter stress while colonizing their host tissue is also thought to contribute to the establishment of a successful infection. The bacterial stringent response is known to trigger a global change of expression in cells of bacteria that can affect the expression of hundreds of genes and increase tolerance to a wide range of environmental stresses. This process is initiated by the rapid synthesis of guanosine tetraphosphate and guanosine pentaphosphate [(p)ppGpp] via the transfer of pyrophosphate from ATP to the 3′-OH group of the ribose in GDP or GTP, respectively (18, 19). The intracellular concentration of (p)ppGpp must therefore be tightly controlled, a process which is mediated by members of the RelA/SpoT homolog (RSH) superfamily, of which there are three types: long RSH enzymes that contain both synthetase and hydrolase domains as well as four characteristic regulatory regions; small molecule synthetases, which only perform a synthetase function; and small molecule hydrolases that contain only a hydrolase domain (20). Although different bacteria utilize a different combination of RSH enzymes, their ultimate function is the same, to regulate the synthesis and degradation of (p)ppGpp, and thereby mediate the adaptive response mechanisms of the cell in response to stress. Bacteria belonging to the Betaproteobacteria and Gammaproteobacteria typically possess two long RSH enzymes, namely RelA and SpoT, while others, including many species from other bacterial classes, usually have only one bifunctional RSH protein, referred to as Rel (21, 22). Typically, (p)ppGpp is produced to cope with stresses, performing regulatory functions by inhibition of *de novo* synthesis of rRNA, tRNA, and ribosomal protein, as well as the activation of amino acid biosynthesis. Meanwhile, the downstream effects on cell physiology can involve altered growth and the initiation of stress responses and survival strategies that all indirectly influence pathogenicity (23–26). However, there is also mounting evidence that (p)ppGpp contributes directly to the virulence of a wide variety of pathogenic bacteria, including *Pseudomonas aeruginosa*, *Salmonella enterica,* and *Mycobacterium tuberculosis*, in which they regulate the transcription of T3Es, flagella-mediated mobility, adhesion, and biofilm formation (27, 28).

Previous research has confirmed that (p)ppGpp plays an important role in regulating key pathogenic factors in plant pathogens such as *Xanthomonas citri* subsp. *citri*, *Pseudomonas syringae*, *Agrobacterium tumefaciens,* and *Erwinia carotovora* subsp. *atroseptica* (29–34). For example, a ppGpp-deficient mutant of *Xanthomonas campestris* pv. *campestris* exhibits reduced production of exopolysaccharides (EPS), biofilm formation, swimming motility, and pathogenicity (34). Our recent studies have established a close relationship between (p)ppGpp and the production of EPS in Cm and demonstrated that (p)ppGpp is an important regulation factor during the infection process. However, it was also noted that the Δ*rel* mutant was still able to effectively colonize the xylem of its tomato host, which raises the question of how critical (p)ppGpp is to the infection process, and what is the extent of its influence over different cellular processes and virulence factors that contribute to the successful infection and colonization (35). The current study was initiated to elaborate on those previous findings and utilize a transcriptome approach in order to identify further genes and processes that ppGpp might influence during the Cm infection process. Additional experiments were conducted to verify the physiological consequences of any virulence factors found to have altered expression.

## RESULTS

### Absence of (p)ppGpp affects expression of a wide range of genes in *C. michiganensis*

**Fig 1:**
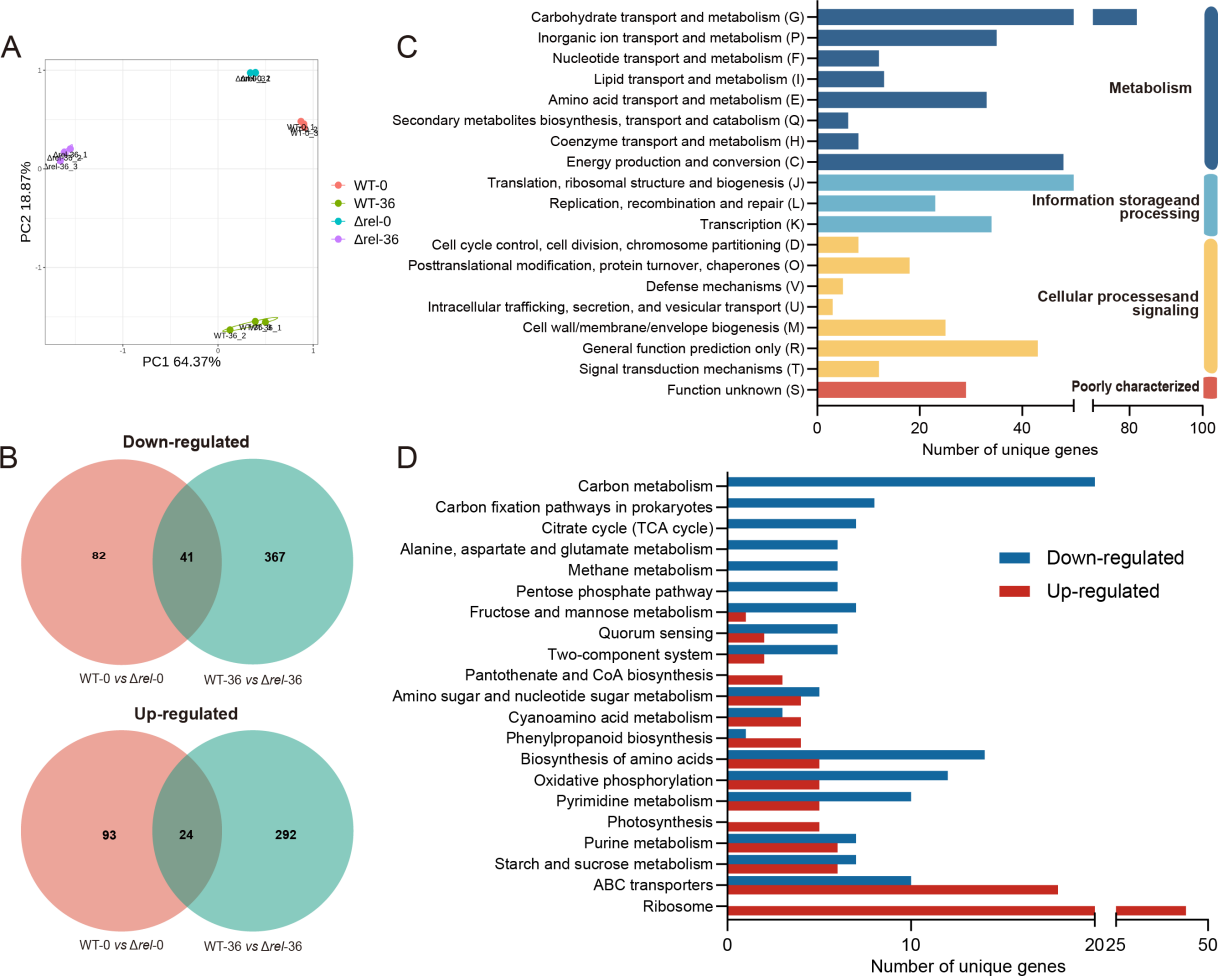
Functional enrichment analysis of DEGs in the Δ*rel* mutant compared to WT *Clavibacter michiganensis*. (**A**) PCA plot indicating the differences in the transcriptome profiles of Δ*rel* and WT at time 0 (−0) and 36 hpi (−36) of a simulated infection system (mM9 supplemented with 10 mL L^−1^ tomato xylem sap). (**B**) Venn diagram illustrating the overlap between the DEGs in the four treatments. (**C, D**) Functional annotation ascribing the specifically expressed genes to different functional categories by reference to the 36 hpi COG and KEGG databases, respectively.

An RNA-seq approach was used to identify specific transcripts regulated by (p)ppGpp by comparing the transcriptome of the wild-type Cm strain BT0505 (WT) to that of a Δ*rel* mutant, which was incapable of producing (p)ppGpp. The investigation also compared gene expression at time 0 and 36 h post-inoculation (hpi) during a simulated infection (modified M9 [mM9] amended with 10% tomato xylem sap). A total of 12 RNA samples (three biological replicates per sample) were evaluated, and after filtering out low-quality reads, the quality control revealed that more than 96% of the original data were of high quality having Q20 values greater than 98% (Table S1). Meanwhile, principal component analysis (PCA) revealed that the three biological replicates of each treatment clustered together to form distinct groups that were significantly different from each other. Indeed, the PCA plot (Fig. 1A; Table S2) not only showed that the Δ*rel* mutant had a significantly different transcriptome compared to the WT, but that there were also significant differences in both strains when comparing the samples collected at time 0 and 36 hpi. However, when the differentially expressed genes (DEGs; assuming a Q-value ≤0.05 and a log2FoldChange ≥1) were examined in greater detail, a small degree of overlap was also seen between the time 0 and 36 hpi treatments (Fig. 1B). For example, of the 409 DEGs found to be up-regulated in Δ*rel* compared to WT, 93 occurred at time 0, 292 at 36 hpi, and 24 in both treatments. Similarly, of the 490 down-regulated genes, 82 were detected at time 0, 367 at 36 hpi, and 41 under both conditions. These results confirmed that (p)ppGpp plays an important role in the infection process as the expression of significantly more genes was affected at the end of the simulated infection process compared to at the beginning. Moreover, of the 2,094 co-expressed genes shared by the 12 samples, approximately 42.93% were found to be differentially expressed in the WT and Δ*rel* samples, indicating an extremely significant regulatory role of (p)ppGpp in the overall metabolism of Cm. Functional annotation was used to gain greater insight into the various cellular processes that might be affected by (p)ppGpp at 36 h mimicking the interaction with tomato, initially by referencing the Cluster of Orthologous Groups (COG) database (Fig. 1C; Table S3). This analysis classified the DEGs into different functional categories, including carbohydrate transport and metabolism, defense mechanisms, translation, ribosomal structure, and biogenesis, which belonged to the broader categories of cellular metabolism, genetic information transmission, and intracellular signaling, as well as genes of unknown functions, and reflected the multiplicity of cellular processes regulated by (p)ppGpp. Meanwhile, the metabolic pathways affected by (p)ppGpp were investigated by Kyoto Encyclopedia of Genes and Genomes (KEGG) annotation (Fig. 1D; Table S4), which revealed that the majority of the genes that were up-regulated in Δ*rel* were involved in metabolic pathways that included cyanoamino acid metabolism, and ribosomal protein synthesis among others, while the downregulated genes were mostly associated with carbon metabolism, oxidative stress, and amino acid metabolism.

### (p)ppGpp is involved in the regulation of multiple pathogenicity factors in *C. michiganensis*

Previous studies have shown that exopolysaccharides (EPS) plays an important role in the pathogenicity of Cm, and that EPS production is significantly reduced in Δ*rel* compared to the WT (35, 36). The synthesis of EPS in Cm seems to be influenced by four gene clusters (EPS I to IV), of which EPS IV is the most well understood, having been found to play an important role in bacterial adhesion and to be the most important gene cluster involved in the production of EPS in Cm grown on solid medium (14). It is therefore interesting to note that of the four EPS clusters, EPS IV exhibited the highest degree of differential expression in Δ*rel*, with the transcriptome heat map showing that 93.33% of its genes exhibited altered expression.

**Fig 2:**
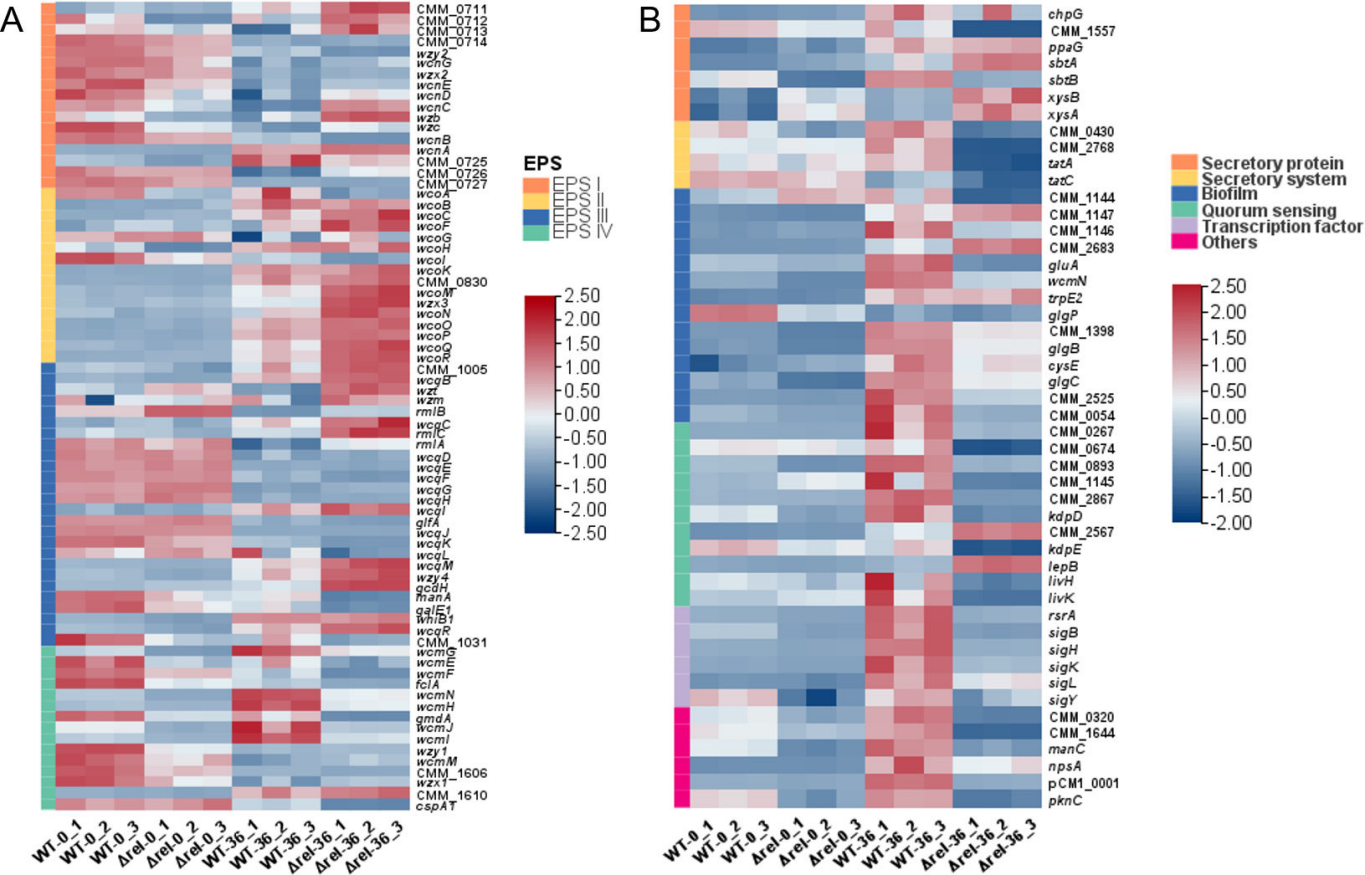
(p)ppGpp regulated the expression of exopolysaccharides (EPS) and pathogenesis-related genes in *Clavibacter michiganensis*. (**A**) The expression profile associated with four EPS synthesis gene clusters. Genes marked in orange, yellow, blue, and green correspond to EPS cluster I–IV. (**B**) The expression profile of pathogenesis-related genes grouped together by COG categories. Genes marked in orange, yellow, blue, green, purple, and pink are associated with secreted protein, secretion system, biofilm, quorum sensing, transcription factor, and other virulence-related genes, respectively. Samples from the (p)ppGpp-deficient mutant (Δ*rel*) were compared to the WT at time 0 (−0) and 36 hpi (−36) of a simulated infection (mM9 supplemented with 10 mL L^−1^ tomato xylem sap).

Although the transcriptome heat map did not find such a consistent pattern of expression for the other three EPS gene clusters in the Δ*rel* mutant, it was interesting to note that a large proportion of the EPS I and EPS III genes were up-regulated at time 0 of the simulated infection process and down-regulated at the end (Fig. 2A; Table S5), while the reverse was true for EPS II. However, the functions of the EPS II and EPS III gene clusters have yet to be determined, while previous research has indicated that EPS I does not in fact play a role in EPS production (14). Nevertheless, the fact that over half of the EPS II genes (56.25%) exhibited a trend of enhanced up-regulation in Δ*rel* at 36 hpi indicates that it is likely that these genes might have an important role in the infection process, even though no difference was found in the capacity of Δ*rel* and the WT to spread and colonization throughout the tomato stems during the *in vivo* pathogenicity assay (Fig. S1).

Unlike the complex double-membrane cell envelope of gram-negative bacteria, gram-positive bacteria have a simple membrane structure. Therefore, it can be speculated that the general membrane transport structures may play a more important role in the pathogenesis of these organisms. It was therefore interesting to note that the transcriptome heat map also revealed quite a distinct trend with regard to genes annotated to the categories of Secretory protein, Secretory system, Biofilm, and Transcription factor (Fig. 2B; Table S6). Although there were no great differences between Δ*rel* and the WT at time 0, at 36 hpi, there was quite a pronounced down-regulation of most of these genes in Δ*rel*, but a dramatic up-regulation in the WT. These results suggested that (p)ppGpp regulates multiple processes associated with pathogenicity, including extracellular protein production, secretion systems, biofilm formation, and transcription factors (Fig. 2B; Table S6). Many of the DEGs identified were found to encode serine proteases or cell-wall degrading enzymes including *chpG*, *ppaG*, *sbtB,* and CMM_1557, which were all downregulated in Δ*rel*. Similarly, genes associated with the secretion system of Cm were also significantly down-regulated, including CMM_0430, 2768, *tatA*, and *tatC*. Other non-enzymic secretions such as polyglycoproteins, lipoproteins, and polysaccharides are also thought to play important roles in the pathogenicity of Cm, and it was interesting to note that genes related to the synthesis and transport of these compounds were significantly downregulated in Δ*rel*, including permeases of the major facilitator superfamily, CMM_1144, asparaginase, CMM_1146, and the glutamate ABC transporter *gluA*. Meanwhile, many genes associated with environmental sensing were found to be up-regulated in the WT at 36 hpi, including those associated with quorum sensing (QS), which is an important regulator of biofilm production, and is known to be a bacterial pathogenicity factor. Although QS has not been fully characterized in Cm, the RNA-seq analysis from the current study indicated that many QS-related genes were significantly down-regulated in Δ*rel*, including two-component regulatory systems such as *kdpD* and *kdpE*, branched-chain amino acid ABC transporters like *livH* and *livK*, and oligopeptide ABC transporters such as CMM_2185, CMM_1587, CMM_1588, and CMM_1589. Furthermore, sigma factors, which are crucial auxiliary proteins of the bacterial RNA polymerase that play a significant role in gene regulation, were also down-regulated in Δ*rel*, including *sigH*, *sigB*, *sigK*, *sigL,* and *sigY*, indicating that the expression of a wide range of specific genes was greatly suppressed compared to WT. Furthermore, a large number of genes with unknown functions, such as CMM_0320 and CMM_1644, were also observed to be downregulated in Δ*rel*, although COG annotation indicated that CMM_0320 is a putative signal peptide hydrolase, and CMM_1644 is a putative protease assembly protein, both of which could be involved in the pathogenicity of Cm.

### (p)ppGpp regulates biofilm formation and extracellular amylase and xylanase activity

**Fig 3:**
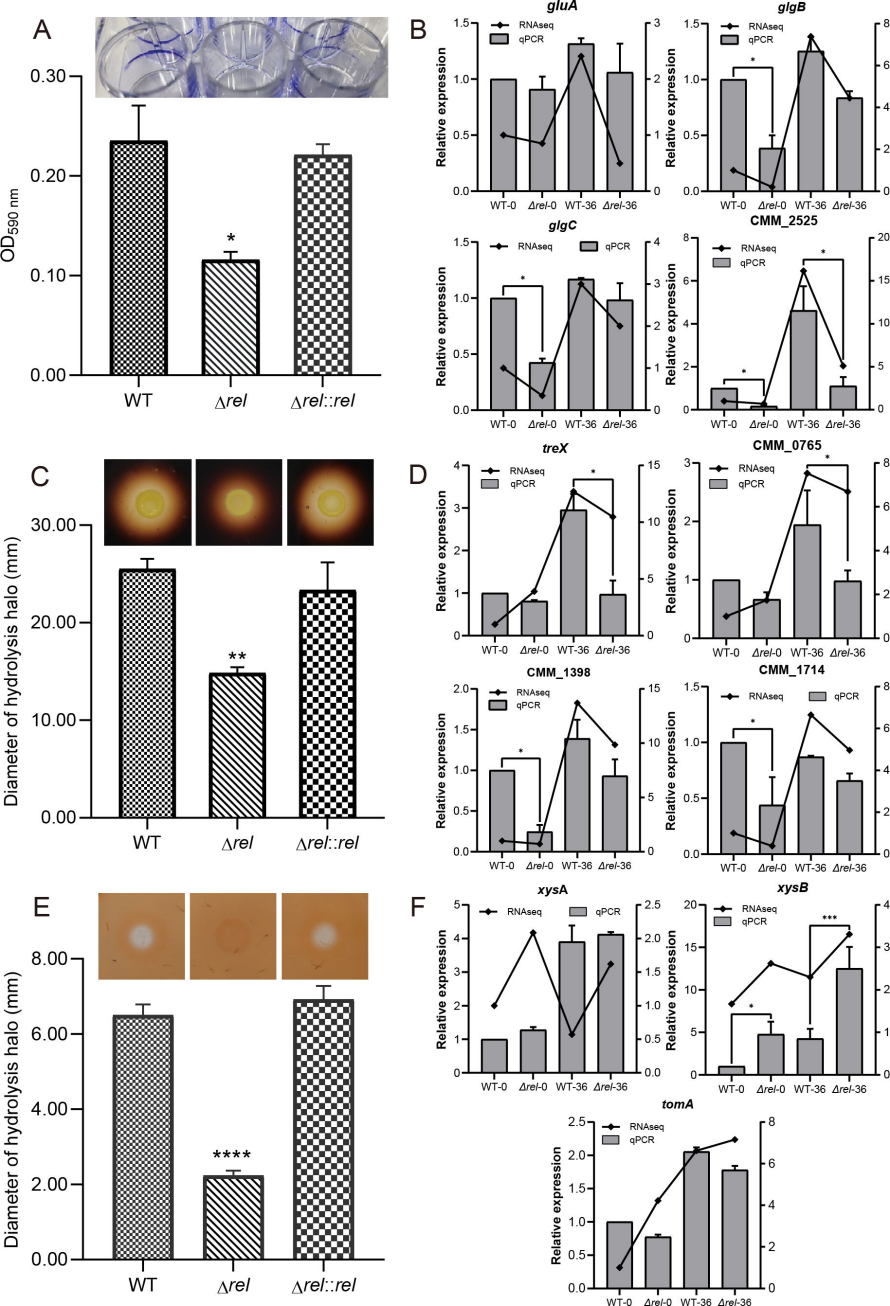
(p)ppGpp has a positive effect on biofilm formation and extracellular enzyme activity in *Clavibacter michiganensis*. (**A**) Biofilm stained *in situ* with crystal violet, and graph showing the corresponding absorbance data (590 nm) when dissolved in absolute ethanol. (**C**) Hydrolysis halos associated with amylase activity, and graph showing the halo diameter. (**E**) Similar hydrolysis halos and measurements corresponding to xylanase activity. Error bars indicate standard deviations from three independent experiments; and asterisks denote statistically significant differences (*P* < 0.01) as determined by one-way analysis of variance (ANOVA) in comparison to the WT sample. (**B, D, F**) Expression levels of genes associated with biofilm formation, as well as amylase and xylanase synthesis and secretion, were compared between WT and Δ*rel* at time 0 and 36 hpi under simulated infection conditions (mM9 medium amended with 10 mL L^−1^ of tomato xylem sap). Error bars indicate standard deviations from three independent experiments, and asterisks denote statistically significant differences (*P* < 0.05) as determined by one-way ANOVA in comparison to the WT sample.

Bacterial biofilms are widely regarded as an important virulence factor in plant pathogenic bacteria, including Cm (37). The yield of biofilm in the WT and Δ*rel* mutant was measured to evaluate whether (p)ppGpp affects its production. Crystal violet staining showed that both the WT and the Δ*rel::rel* complementation strain produced a discernible quantity of biofilm on the inner wall of the 24-well plates, while that produced by the Δ*rel* was barely visible (Fig. 3A). These results were confirmed by the quantitative analysis, which confirmed that the Δ*rel* mutant produced approximately 50% less biofilm than the WT (Fig. 3A). Furthermore, qPCR analysis found that the expression of *gluA*, *glgB*, *glgC*, and CMM_2525, key genes associated with biofilm synthesis in Cm, were significantly downregulated in Δ*rel* (Fig. 3B), which was consistent with the results of RNA-seq analysis, and provided further evidence that (p)ppGpp plays an important role in regulating biofilm synthesis in Cm.

Hydrolase enzymes such as cell wall-degrading enzymes are also important pathogenic factors in plant pathogenic bacteria. The plate assays in the current study demonstrated that (p)ppGpp also plays an important role in this process in Cm, as the Δ*rel* mutant produced much diminished hydrolysis halos (14.83 ± 1.00 mm) compared to either the WT or the Δ*rel::rel* complementary strain (25.50 ± 1.00 mm and 23.33 ± 4.50 mm, respectively) when grown on media containing soluble starch in conjunction with I_2_/KI straining (Fig. 3C). These results were consistent with the RNA-seq data which revealed that genes associated with amylase production and secretion such as *treX*, CMM_0766, CMM_1389, and CMM_1714 were down-regulated in Δ*rel*, an observation which was confirmed by qPCR (Fig. 3D).

Similar analysis revealed that the Δ*rel* mutant also exhibited a reduced capacity to hydrolyze xylan (Fig. 3E) as indicated by the diminutive halo (2.23 ± 0.18 mm) produced on media containing xylan and stained with Congo red in comparison to the WT and Δ*rel::rel* (6.50 ± 0.41 mm and 6.92 ± 0.51 mm, respectively). This was an interesting observation as the transcriptome analysis had indicated that the *xysB* gene encoding the xylanase in Cm was actually significantly up-regulated in Δ*rel*. Although qPCR confirmed this result, it also demonstrated that the expression of alternative xylanase enzymes encoded by *xysA* and *tomA* did not differ greatly between Δ*rel* and the WT (Fig. 3F). Indeed, the expression of *xysA* was found to be upregulated over the entire duration of the infection process when assessed *in vivo* in tomato seedlings, beginning at 3 hpi and fluctuating thereafter until forming a huge peak at 168 hpi with no significant difference between the two strains. In contrast, the *in vivo* expression of *xysB* was found to increase gradually in Δ*rel* until forming a large peak at 168 hpi, while expression remained consistently low in the WT until also forming a dramatic peak at 168 hpi (Fig. S2). Taken together, these results indicate that (p)ppGpp does play a key role in mediating extracellular xylanase activity in Cm, but that this process likely involves regulating the secretion of xylanase enzymes rather than their synthesis.

However, (p)ppGpp did not seem to play a similar role in the regulation of extracellular cellulase activity in Cm, as no significant differences were found between the Δ*rel* mutant and either the WT or the Δ*rel::rel* complementation strain in similar plate assays assessing the hydrolysis of carboxymethyl cellulose (Fig. S3).

Taken together, these results indicate that (p)ppGpp plays a significant role in various processes associated with the pathogenicity of Cm, including biofilm formation and the production and secretion of certain hydrolase enzymes, although the precise mechanism of regulation might differ in each case.

### The Vatr1 transcription factor negatively regulates the expression of *xysB*

**Fig 4:**
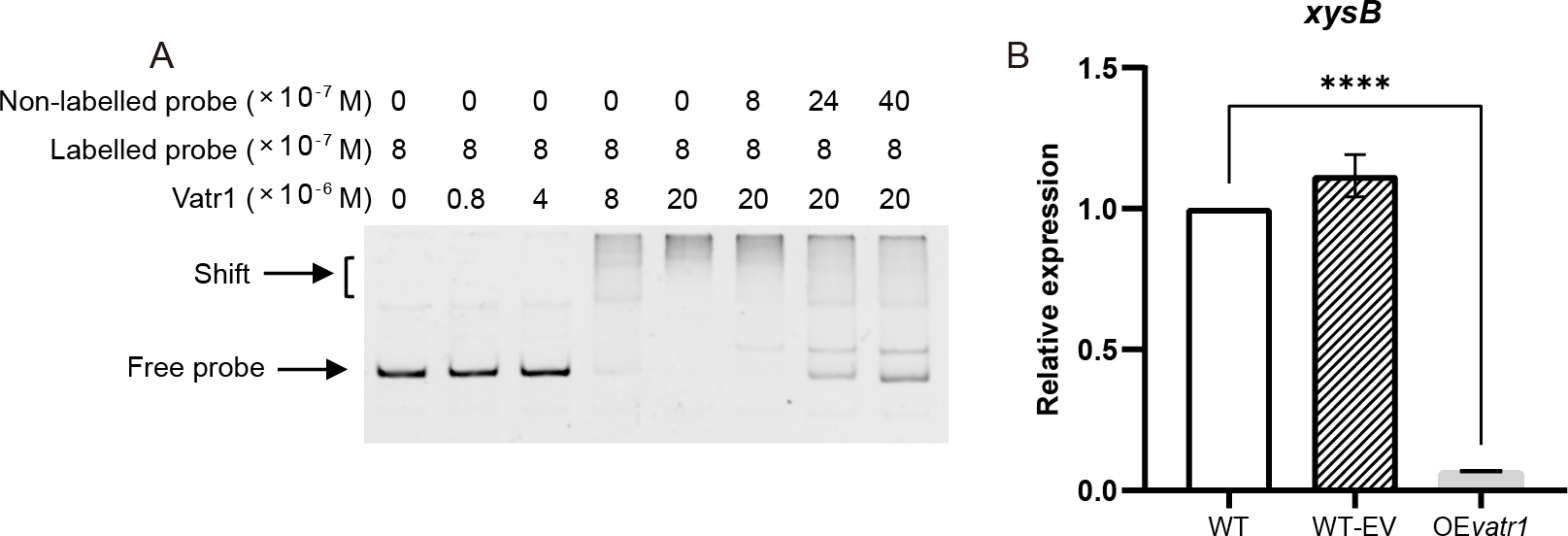
The Vatr1 transcription factor suppresses *xysB* expression in *Clavibacter michiganensis*. (**A**) EMSA in which 8 × 10^−7^ M samples of fluorescein amidite (FAM)-labeled probes consisting of the *xsyB* promoter DNA (374 bp) were incubated with increasing concentrations (0.8–20 × 10^−6^ M) of Vatr1. (**B**) Relative expression of *xysB* in a (p)ppGpp-deficient mutant (Δ*rel*) and the wild-type parental strain (WT), as well as the WT transformed with either an empty vector (WT-EV), or a *vatr1* overexpression vector (OE*vatr1*). Data represent the mean ± SD, with two or four asterisks indicating different degrees of significance (*P* < 0.01 and 0.0001, respectively), according to a one-way analysis of variance in comparison to the WT sample.

The Vatr1 transcription factor, which belongs to the TetR family of regulatory factors, is known to play an important role in the pathogenicity of Cm, and the results of the transcriptome analysis conducted in the current study suggested that it might be involved in the regulation of *xysB* (17). Electrophoretic mobility shift assay (EMSA) using a 374 bp DNA fragment corresponding to the predicted *xysB* promoter sequence confirmed an interaction with the Vatr1 protein as the migration of the promoter DNA through a polyacrylamide gel was impaired (Fig. 4A). Furthermore, the specificity of the interaction was confirmed as competition with an unlabeled probe reduced the effectiveness of the mobility shift.

qPCR confirmed the suppression of *xysB* expression by Vatr1 *in vivo*, as overexpression of the *vatr1* gene using a modified shuttle vector (pHN216-*J23119*-vatr1) resulted in significantly reduced *xysB* expression in Cm (Fig. 4B). Furthermore, *xysB* expression was found to be significantly up-regulated in the Δ*rel* mutant compared to the WT (Fig. 3F), indicating that (p)ppGpp and Vatr1 act cooperatively to inhibit the expression of *xysB in vivo*.

### ppGpp and Vatr1 cooperatively inhibit *xysB* transcription

**Fig 5:**
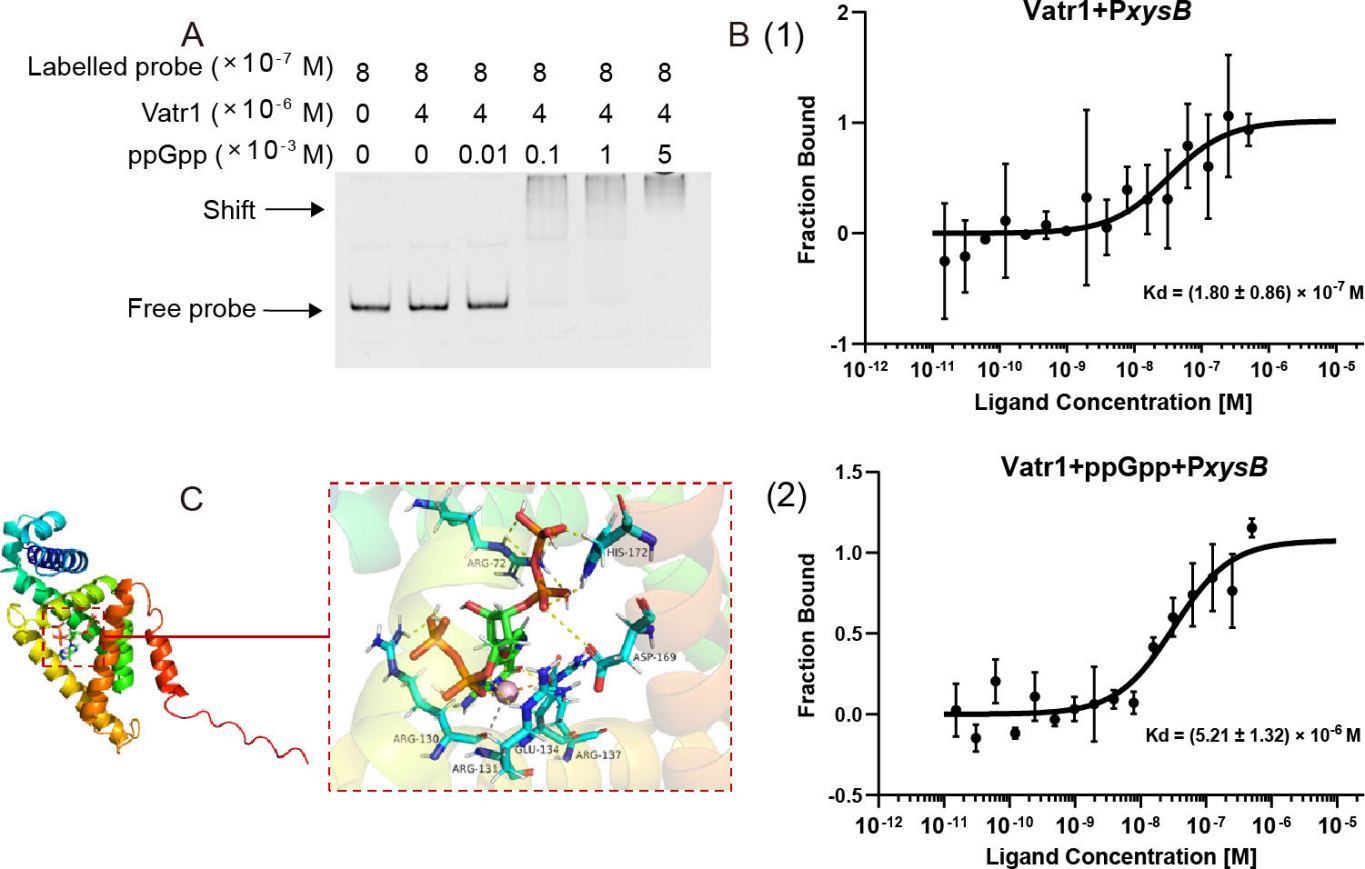
The presence of ppGpp enhances Vatr1-mediated suppression of *xysB* transcription. (**A**) EMSA in which 8 × 10^−7^ M samples of fluorescein amidite (FAM)-labeled probes consisting of *xsyB* promoter DNA (374 bp) were incubated with 8 × 10^−6^ M Vatr1 and increasing concentrations (0–5 × 10^−3^ M) of ppGpp. (**B**) Microscale thermophoresis analysis in which the affinity of Vatr1 with *xysB* promoter was assessed in either the absence or presence of ppGpp. (**C**) Molecular model illustrating potential interactions between Vatr1 and ppGpp in the formation of the Vatr1-ppGpp-Mg^2+^ complex. The zoomed-in view shows a detailed view of how the guanine and ribose groups (green) and phosphate groups (orange) of ppGpp are coordinated in the allosteric site, as well as the Vatr1 side chains that form the metal-acceptor interactions via Mg^2+^ (pink ball), and the hydrogen bonds, attractive charge, and metal-acceptor (yellow, gray, and orange dashed lines) that cause a conformational change in the Vatr1 structure.

The EMSA analysis was performed to determine whether (p)ppGpp interacts with Vatr1 and thereby affects its binding to the *xysB* promoter. The results confirmed this hypothesis, as the addition of ppGpp seemed to increase the strength of the mobility shift in a dose-dependent way (Fig. 5A). The nature of the ppGpp-Vatr1 interaction was further clarified by microscale thermophoresis (14), which revealed that the dissociation constant (*K_d_*) of the *xysB* promoter and Vatr1 was significantly lower when ppGpp (3 × 10^−2^ M) was present (5.21 ± 1.32 × 10^−9^ M) compared to when it was absent (18.02 ± 8.63 × 10^−9^ M), indicating that ppGpp increased the affinity of Vatr1 to its target DNA and caused it to bind to it more strongly (Fig. 5B).

Molecular docking analysis was used to investigate the precise biochemical basis for the interaction between ppGpp and Vtar1. The results indicated that ppGpp forms metal-acceptor interactions with the GLU134 and ARG130 residues of the Vatr1 protein via Mg^2+^, as well as hydrogen bonds with ARG72, HIS172, ASP169, ARG137, ARG131, and ARG130 (Fig. 5C). It was predicted that, in turn, these interactions induce a conformational change in the helix-turn-helix domain of the Vatr1 protein, thereby increasing its affinity for the *xysB* promoter and enhancing suppression of *xysB* transcription.

### Low expression of the Tat system inhibits secretion of XysB

**Fig 6:**
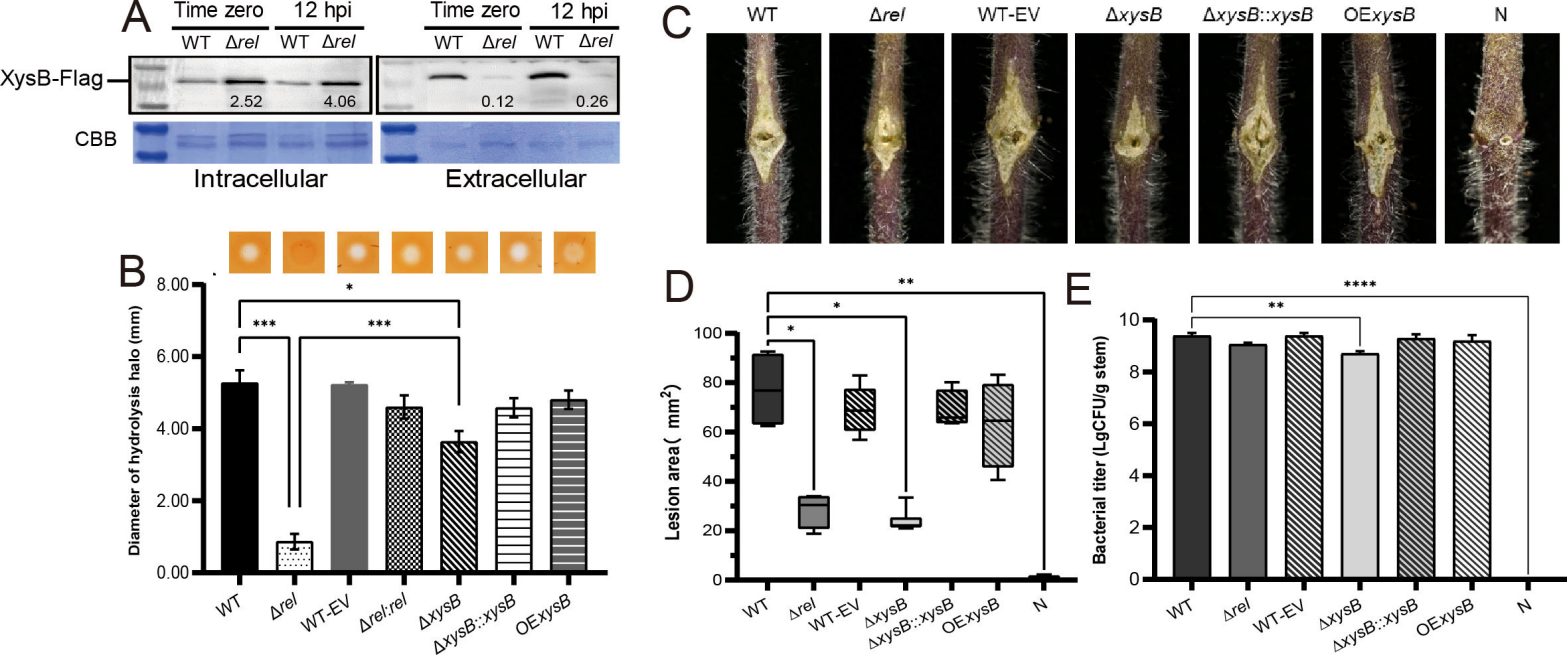
The role of XysB in pathogenicity is inconsistent with its expression level in *Clavibacter michiganensis*. (**A**) Western blot analysis comparing the intracellular and extracellular accumulation of the XysB protein in both a (p)ppGpp-deficient mutant (Δ*rel*) and the WT at time 0 and 12 hpi of a simulated infection system (mM9 supplemented with 10 mL L^−1^ tomato xylem sap treatment). The molecular weight marker lane was digitally added during chemiluminescence detection using the Azure C600 imager for reference. (**B**) Hydrolysis halos associated with the xylanase activity of Δ*rel* and the WT, as well as a Δ*xysB* mutant, two complementation strains (Δ*rel::rel* and Δ*xysB::xysB*), an *xysB* overexpression strain (OE *xysB*), and the transformation control strain (WT-EV), and graph showing the halo diameters. (**C**) Symptoms resulting from prick inoculation of tomato plants (20 days post-inoculation) with various Cm strains. (**D**) Graphs indicating the lesion size measured by ImageJ software. (**E**) Corresponding bacterial cell titers from each lesion. Data represent the mean ± SD; with one, two, three, or four asterisks indicating different degrees of significance (*P* < 0.05, 0.01, 0.001, and 0.0001, respectively) according to a one-way analysis of variance in comparison to the WT sample.

Having established that the expression of *xysB* was up-regulated in the Δ*rel* mutant, but that extracellular xylanase activity was reduced, further experiments were designed to investigate the cause of these contradictory findings. Western blotting was used to compare XysB protein samples from Δ*rel* and the WT at time 0 and 12 hpi during the simulated infection, and collected from both harvested cells and the growth medium. The results revealed that the XysB protein accumulated within the cells of the Δ*rel* mutant and was only secreted to the growth medium in very small amounts. Meanwhile, in contrast, relatively low levels of XysB were detected in the cells of the WT, but very high levels were detected in the growth media (Fig. 6A). Indeed, the cells of the mutant accumulated 2.52- and 4.06-fold more protein than the WT at time 0 and 12 hpi, respectively, while the amount of XysB in the media was reduced to 0.12- and 0.26-fold that of the WT. Additional xylanase assays were also performed in which a XysB-deficient Δ*xysB* mutant was compared to the WT and the Δ*rel* mutant. Although it was found that the Δ*xysB* mutant had significantly (*P <* 0.05) reduced xylanase activity (27.3% less) compared to the WT, its residual activity was still significantly greater (*P <* 0.001) than that of the Δ*rel* mutant, indicating not only that XysB was not the primary enzyme responsible for xylanase activity in Cm under all circumstances, but that (p)ppGpp also seemed to be required for the secretion of the alternative xylanase enzymes (Fig. 6B). Meanwhile, *in vitro* pathogenicity assays indicated that extracellular xylanase activity played a significant role during the infection process, with both mutants producing smaller lesions (Fig. 6C). However, it was interesting to note that the size of lesions produced by the two mutants did not differ significantly, and that only the Δ*xysB* mutant exhibited a reduced cell titer in homogenized stem samples. Furthermore, it was also noted that overexpression of XysB resulted in neither an increase in xylanase activity nor pathogenicity in the WT (Fig. 6B and C).

**Fig 7:**
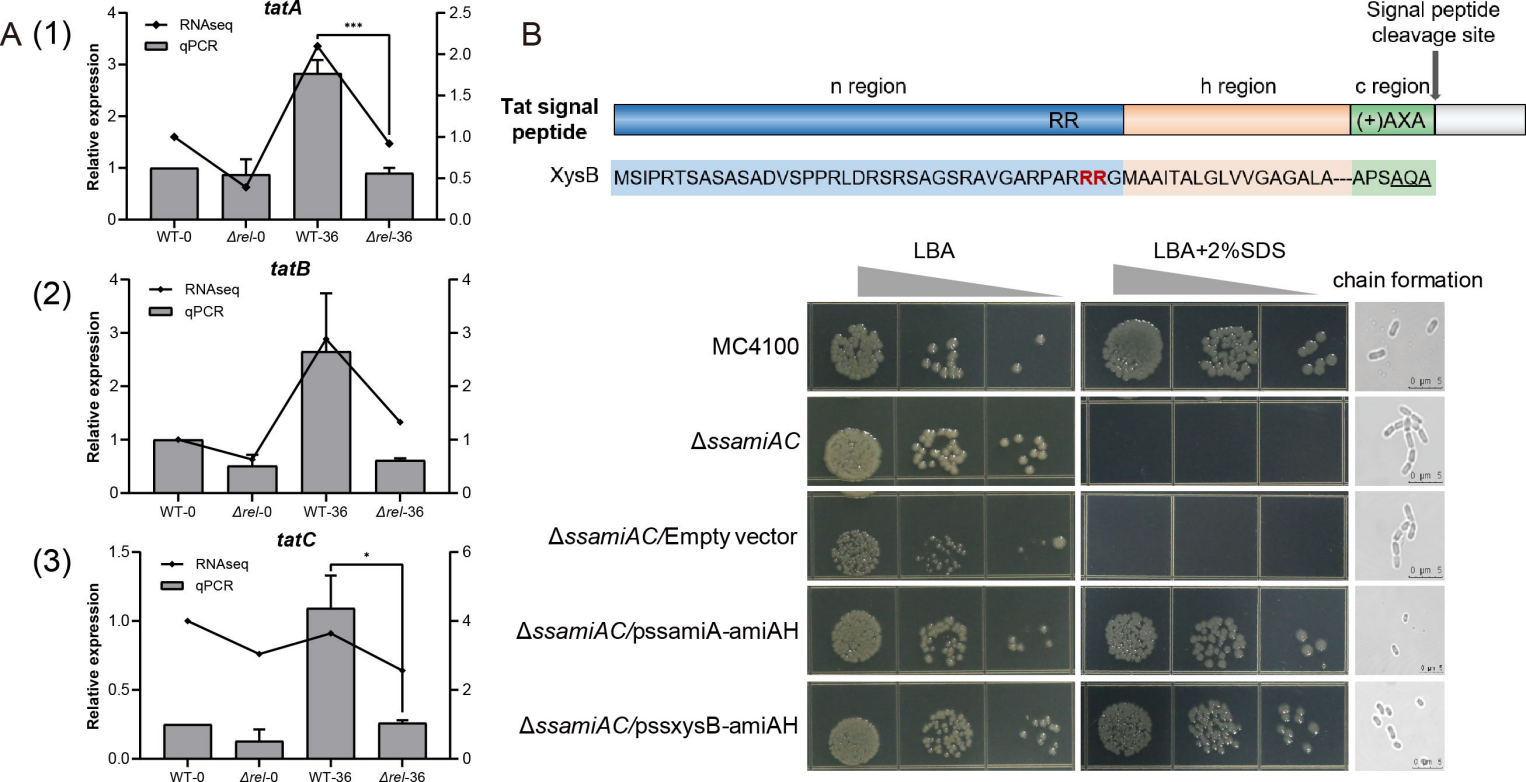
Inhibition of the Tat system is the likely cause of reduced extracellular xylanase activity in a Δ*rel* mutant of *Clavibacter michiganensis*. (**A**) Expression levels of the genes (*tatA*, *tatB*, and *tatC*) associated with the Tat system were assessed in the WT and a (p)ppGpp-deficient mutant (Δ*rel*) at time 0 and 36 hpi of a simulated infection system (mM9 amended with 10 mL L^−1^ tomato xylem sap). (**B**) The figure above represents predicted signal peptides of xylanase enzymes XysB produced by SignalP-6.0 software. The figure below is an amidase reporter assay, in which a Δ*ssamiAC* mutant of *E. coli* lacking a functional amidase enzyme was transformed with constructs fusing the sequence encoding the XysB signal peptides with the *amiA* gene (pssXysB-AmiAH), with a native *amiA* construct (pssAmiA-amiAH) and an empty vector (pSD72) being used as the positive and negative controls, respectively. The wild-type parental strain (MC4100) is included for comparison. The formation of colonies on LBA amended with 2% SDS validated Tat-compatible signal peptides that can rescue the Δ*ssamiAC* phenotype, which is characterized by a lack of growth on media containing SDS and the production of chains of linked bacterial cells when viewed by microscopy (Zeiss LSM 800 microscope). Gray sloping wedges indicate the increasing dilution factor of inoculum (10^−3^–10^−5^), and scale bars indicate 5 µm.

Previous research has shown that (p)ppGpp can regulate bacterial pathogenicity and physiological adaptation via regulation of the T3SS and T6SS translocation pathways of gram-negative bacteria (38–40). Meanwhile, the Tat pathway is utilized for the transport of folded proteins across the cytoplasmic membrane in most bacteria (41). The qPCR analysis conducted in the current study found a significant reduction in the expression levels of genes associated with the Tat system (*tatA*, *tatB*, and *tatC*) in the Δ*rel* strain, a trend that was particularly notable at 36 hpi of the simulated infection, when the expression of these genes in the WT was dramatically up-regulated (Fig. 7A). Meanwhile, a subsequent bioinformatics analysis suggested that the XysB was a highly probable candidate substrate for the Tat transport system, with protein predicted to have a signal peptide containing adjacent arginine residues (Fig. 7B). The *Escherichia coli* amidase reporter assay used in a previous study to identify substrates of the Tat system in other bacteria, such as *Brucella melitensis*, was utilized to assess the compatibility of the XysB signal peptide (42). In this assay, a Δ*ssaimAC* strain, which lacks the *amiA* and *amiC* signal sequences required for the secretion of a functional amidase enzyme via the Tat system, and is therefore unable to form colonies on media containing SDS, is used to validate putative Tat signal peptides via transformation with amidase fusion constructs. In the current study, transformation with recombinant plasmid that fused the sequences encoding the XysB signal peptide with the *amiA* gene (pssXysB-AmiAH) was found to rescue the Δ*ssaimAC* mutant as effectively as the positive control vector expressing a native amidase construct (pssAmiA-AmiAH), as shown in Fig. 7C. All of the transformed Δ*ssaimAC* strains, except the negative control, which was transformed with an empty vector, were found to form colonies on Luria-Bertani agar (LBA) containing 2% SDS, and none produced the characteristic chains of bacterial cells associated with the Δ*ssaimAC* phenotype when examined by microscopy. Meanwhile, the other Cm xylanases, XysA and TomA, were also identified to be translocated by the Tat system (Fig. S4). These results not only confirmed that the XysB signal peptide was compatible with the Tat system but also provided an explanation as to why XysB was up-regulated in the Δ*rel* mutant, yet still had a lower extracellular xylanase activity than the Δ*xysB* mutant, and why the XysB peptide was found to accumulate within the Cm cells. The reason is that the absence of (p)ppGpp in Δ*rel* suppressed the expression of the *tat* genes and inhibited the formation of a functional Tat system, thereby preventing xylanase from being secreted.

## DISCUSSION

Adaptation to the environment during the infection process requires flexible regulatory networks and signaling mechanisms to allow pathogenic bacteria to coordinate their physiological processes and virulence factors. It is well known that the alarmone (p)ppGpp enables bacteria to alter their physiology in order to accommodate fluctuating nutrient supplies and environmental stresses by the suppression or activation of transcription factors and the expression of cellular enzymes. Many reports have highlighted the crucial role of (p)ppGpp in the virulence of gram-negative bacteria, including the regulation of T3SS, flagella-mediated mobility, type IV pili, and biofilm formation to name but a few (29, 31, 43). The underlying mechanisms responsible for these regulatory effects have been extensively investigated in *E. coli*, and it appears that (p)ppGpp binds to two sites on its RNA polymerase to either stimulate or suppress transcription (44, 45). Other regulatory mechanisms have also been documented in other species of bacteria. For example, (p)ppGpp has been shown to bind to the MglA-SspA heterodimer to facilitate the anchoring of the unique transcriptional activator PigR to specific virulence promoters, resulting in the enhanced expression of pathogenicity island genes in *Francisella tularensis* (24). However, the role of (p)ppGpp in gram-positive plant pathogens is still poorly understood. In *C. michiganensis*, it has been reported that a ppGpp-deficient *rel* knockout mutant (Δ*rel*) derived from the wild-type strain BT0505 (WT) exhibited reduced EPS production and virulence even though its capacity to colonize tomato stem tissue was not significantly affected at 21 days post-inoculation (dpi) (36). These results were confirmed in the current study with no differences being found between the cell titers of Δ*rel* and the WT in stem samples collected from areas near the inoculation site at different time points during the infection process (Fig. S1). Similar results were also observed in *Mycobacterium tuberculosis* where (p)ppGpp seemed to have little effect on the colonization of lung tissue during the early stages of infection, although there was a significant correlation at 5 to 7 weeks (46). However, they do contrast to most studies of gram-negative bacteria, in which the level of (p)ppGpp is positively correlated with the capacity to colonize host tissue (29–31, 43). Based on the results of the current study, it appears that the relationship between (p)ppGpp and the virulence of gram-positive bacteria could be more complex than for gram-negative species.

Transcriptome analysis was utilized to perform an in-depth investigation of the regulatory relationship between (p)ppGpp and the pathogenicity of Cm. Although *in vivo* samples were considered, the amount of Cm in tomato stem samples was insufficient to yield enough RNA for the analysis. Instead, the infection process was simulated *in vitro* using Δ*rel* and WT cells cultured in mM9 media amended with 10 mL L^−1^ tomato xylem sap. The expression of the *ppaA* and *pelA* genes was monitored at various time points post-inoculation and found to peak at 24 h and slightly decrease at 36 h, while the expression of *celA* continued to accumulate throughout the whole period of incubation (Fig. S5). Consequently, time 0 and 36 hpi were selected as the most appropriate sample times for the RNA-seq analysis. Comparison of the resulting transcriptome data revealed the profound impact of (p)ppGpp on gene expression in Cm as the genes associated with numerous cellular processes exhibited significantly altered expression in the Δ*rel* mutant including those affecting the production and transport of exopolysaccharides, the formation of biofilm, exocytosis hydrolase synthesis, transcription factors, and secretion systems. Further experiments, including qPCR and biochemical assays, confirmed the changes in EPS production, biofilm formation, and the extracellular activity of amylase and xylanase enzymes in the (p)ppGpp-deficient Δ*rel* mutant, establishing a critical link between (p)ppGpp and key pathogenesis factors in Cm.

It has been noted that plant pathogenic bacteria often produce multiple xylanase enzymes that have different substrate specificity and play an important role during the infection process. In Cm, there appears to be three xylanase genes, *xysA*, *xysB*, and *tomA*, and it was interesting to note the difference in their expression profiles in Δ*rel* and WT (Fig. 3F). Although all three were found to be up-regulated in both strains at 36 hpi, the expression of XysB was different from that of XysA and TomA, having an almost negligible level of expression in the WT at time 0, which only increased at 36 hpi to approximately the baseline level observed in the Δ*rel* mutant at time 0. Furthermore, these results suggest that (p)ppGpp has an inhibitory effect on the expression of *xysB*, which does not occur with *xysA* and perhaps indicates the suppression of XysB activity during the early stages of infection. Most known xylanases are classified into two GH families, 10 and 11, based on the amino acid composition of their xylanase catalytic region, and the sequence of their hydrophobic clusters (47, 48). The three homologous xylanase enzymes of Cm, XysA, XysB, and TomA, belong to the GH10 family and exhibit the typical (β/α) barrel structure. However, XysB differs from XysA and TomA in having a higher molecular weight of 71.32 kDa, and in addition to the GH10 domain, a substrate-specific galactose-binding-like domain, which is known to enhance substrate-binding efficiency to proteases, and a cell-adhesive HYR (HYalin Repeat) domain, both of which suggest that XysB has a stronger substrate-binding affinity for the complex xylan polymers found in plant cell walls (49, 50). In contrast, TomA is smaller at 58.18 kDa and is characterized by two identical immunoglobulin-like folds at its C-terminus, while XysA is smaller still at 46.87 kDa and contains only a single GH10 domain. Despite their shared GH10 family lineage, the low sequence homology of XysA, XysB, and TomA suggests that they have distinct roles during the infection process, while the results of the current study indicate that (p)ppGpp only affects the transcription of *xysB*.

Previous research has shown that the hemicellulose polysaccharides xylan and xyloglucan are frequently acetylated to regulate plant developmental processes, and there is considerable evidence that diminished cell wall acetylation or disruption can activate plant defense responses to pathogen invasions (51–54). Further investigation conducted by EMSA confirmed an interaction between (p)ppGpp and the transcription factor Vatr1, which resulted in enhanced suppression of *xysB* expression. Vatr1 is a transcription factor belonging to the TetR family, which commonly functions as a repressor and occurs in a wide range of bacteria (55). The observation that (p)ppGpp interacts with Vatr1 to enhance suppression of the XysB xylanase, particularly at the beginning of the infection process, could be indicative of a strategy to avoid activation of the xylan-mediated defense response in its plant host.

Having established that (p)ppGpp tightly regulated the expression of the *xysB* gene, it was notable that its up-regulation in the mutant did not result in an increase in its extracellular xylanase activity but, on the contrary, a marked decrease. Western blotting revealed an accumulation of intracellular XysB protein, indicating that the secretion of XysB was not occurring. Meanwhile, the transcriptome analysis revealed that genes associated with the Tat system were down-regulated in the Δ*rel* mutant, indicating that (p)ppGpp was essential for the correct function of this transport system in Cm. These findings were further confirmed by qPCR and amidase assays, which demonstrated that the XysB and its signal peptides were compatible with the Tat system. However, further attempts to investigate the Tat system by knocking out the *tatA* and *tatC* genes in Cm by homologous recombination were unsuccessful. Nonetheless, taken together, these results provide great insight into the pathogenicity of Cm, indicating that (p)ppGpp plays a dual role in regulating its xylanase activity, not only by mediating the expression of the *xysB* gene, but more critically by controlling the secretion of both the XysB, XysA, and TomA proteins. Previous studies have demonstrated that the Tat-secreted proteins contribute to the full virulence of gram-positive pathogenic bacteria (56–58). A similar result has also been found for the gram-positive soil bacterium *Streptomyces lividans*, which secretes xylanase enzymes via the Tat system, which is in contrast to gram-negative *Xanthomonas* spp. that secrete them via the T2SS system after they have been transported across the inner membrane by the Sec system (59–62). It is well-established that the Tat and Sec secretion pathways of Cm operate independently and have distinct functions. Several serine proteases like ChpG and PpaA, which were found to be up-regulated by (p)ppGpp in Cm, were also predicted to have Sec signal peptides (Fig. S6). Meanwhile, the discovery that (p)ppGpp regulates the Tat system in Cm has greater significance, as this would also affect the secretion of other pathogenicity factors, including expansin and amylase enzymes (Fig. S4).

**Fig 8:**
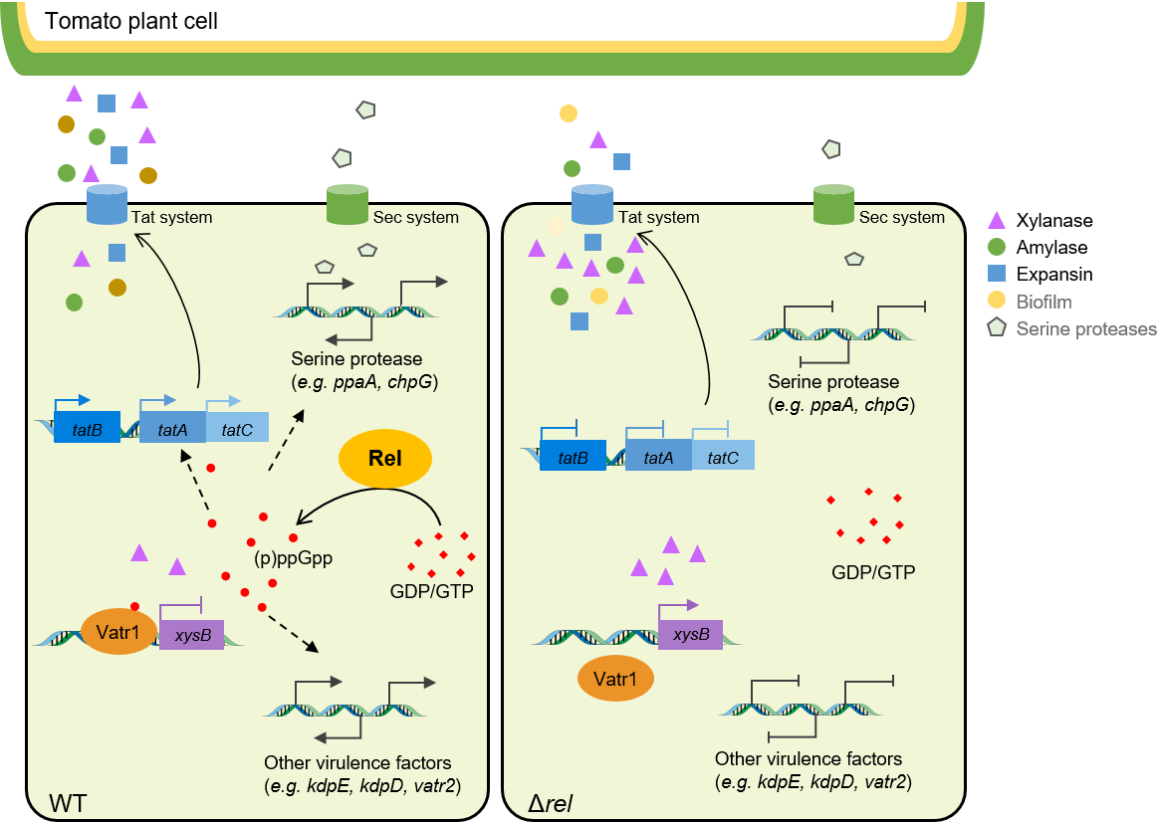
Schematic overview illustrating (p)ppGpp-mediated regulation of virulence factors in *Clavibacter michiganensis*. During the pathogenic process of *Clavibacter michiganensis* infecting tomato stems, the Rel enzyme catalyzes the synthesis of (p)ppGpp by transferring pyrophosphate from ATP to GDP or GTP. (p)ppGpp acts as a global regulator, enhancing the transcription of genes associated with biofilm formation, serine proteases, and additional virulence factors. In addition, (p)ppGpp increases the expression of the genes required for the Tat system, which facilitates the secretion of XysB and other cell-wall digesting enzymes and virulence factors. However, at high intracellular concentrations, (p)ppGpp amplifies the inhibitory effect of the transcription regulator Vatr1 on the expression of *xysB*. In the Δ*rel* mutant, which is incapable of producing (p)ppGpp, *xysB* expression is significantly up-regulated, while the transcription of genes encoding the Tat system is markedly reduced. This results in the intracellular accumulation of the XysB xylanase and a corresponding decrease in extracellular enzyme activity.

Previous research has shown that (p)ppGpp can regulate the expression of the type IV secretion system by controlling the expression of *virB* in the mammalian pathogens such as *Brucella melitensis*, *Brucella suis,* and *Bartonella henselae*, and that a (p)ppGpp-mediated stringent response can activate the T2SS and T3SS to initiate pathogenesis in the gram-negative plant pathogens *Xanthomonas citri* and *Erwinia amylovora* (31, 38, 39, 63). Here, we confirmed that (p)ppGpp has a critical role in the pathogenicity and infection process of the gram-positive species *C. michiganensis* by regulation of the Tat system. Indeed, the results of the current study have greatly expanded our understanding of the crucial role that (p)ppGpp plays in the overall pathogenicity of this representative gram-positive plant pathogen, as summarized in Fig. 8.

## MATERIALS AND METHODS

### Bacterial strains and culture conditions

The strains, plasmids, and primers used in the current study are listed in Tables S7 and S8. The wild-type parental Cm isolate BT0505 (WT) as well as its mutant derivatives were routinely cultured on Luria-Bertani agar (LBA; 10 g L^−1^ tryptone, 5 g L^−1^ yeast extract, 5 g L^−1^ NaCl, and 16 g L^−1^ agar) at 28°C for 3 days. Meanwhile, modified M9 (mM9) medium (6 g L^−1^ Na_2_HPO_4_ 12H_2_O, 3 g L^−1^ KH_2_PO_4_, 1 g L^−1^ NH_4_Cl, 0.5 g L^−1^ NaCl, 1 × 10^−3^ M MgSO_4_ 7H_2_O, 1 × 10^−5^ M CaCl_2_ 2H_2_O, 2 g L^−1^ glucose, 200 mg L^−1^ methionine, 200 mg L^−1^ thiamine, 20 mg L^−1^ nicotinic acid, and 16 g L^−1^ agar) was used for some of the experimental assays. For transformation assays, sorbitol broth (SB; 10 g L^−1^ trypsin, 5 g L^−1^ yeast extract, 4 g L^−1^ NaCl, 91 g L^−1^ D-sorbitol, 2 × 10^−2^ M MgCl_2_ 6H_2_O, 2 × 10^−2^ M CaCl_2_ 2H_2_O) or SBA (SB supplemented with 16 g L^−1^ agar) was used (15). The *E. coli* strains used in the cloning were grown on LBA medium at 37°C overnight. When required, Cm liquid cultures were prepared in Luria-Bertani (LB) and incubated at 28°C, with shaking (150 rpm) for 20–24 h, while *E. coli* were prepared in LB at 37°C with shaking (200 rpm) for 12 h. If needed, selective antibiotics were used at the following concentrations: chloramphenicol, 10 µg mL^−1^; kanamycin, 50 µg mL^−1^; neomycin, 50 µg mL^−1^; ampicillin, 100 µg mL^−1^, while 100 µg mL^−1^ 5-flucytosine (5-FC) was used for negative selection.

### Cloning and transformation

The Cm Δ*xysB* mutant was generated using an unmarked CRISPR/Cas9-mediated approach as previously described (64). Briefly, the knockout vector pHN216*xysB*sg1779-codAupp was generated from the pHN216-katA-codAupp template plasmid by replacing the *katA* donor DNA and signal guide RNA (sgRNA) with flanking regions from the *xysB* gene obtained by PCR with three primer sets (xysBupF/xysBupR, xysBdownF/xysBdownR, and CxysBsgRNAF/CxysBsg1977revR), which were designed following the instructions detailed on the EuPaGDT website (http://grna.ctegd.uga.edu/). The resulting knockout vector was then transformed into the WT by electroporation (1.8 kV, 15 ~ 18 ms). Individual clones were selected and cultured in LB for 24 h, before being diluted (10^−4^ or 10^−5^) and plated on mM9 agar supplemented with 100 µg mL^−1^ 5-FC. A separate 100 µL aliquot was reserved and used to re-inoculate fresh LB, which was incubated for a further 24 h, upon which the process was repeated. This procedure was conducted four times, before individual colonies were picked for verification by DNA sequencing to confirm the successful generation of the Δ*xysB* mutant.

The Vatr1 overexpression strain (OE*vatr1*) was created by transforming BT0505 with the pHN216-*J23119*-vatr1 vector by electroporation. The plasmid itself was produced by introducing the full-length *vatr1* coding region, which was amplified using the oevatr1-F/oevatr1-R primer set, into the pNH216 vector containing an enhanced *J23119* promoter.

The pHN216-xysB-Flag complementation vector was generated by introducing the full-length *xysB* gene including its native promoter into pNH216 using In-Fusion cloning and the xysBNPF/FxysBR primer set, which also added a 3×Flag to the C-terminal of the translated protein. The resulting vector was electrophorated into the Δ*rel* mutant for complementation and WT for overexpression.

The Vatr1 expression vector was constructed by amplifying the full-length *vatr1* coding region with the Vatr1GST-F/Vatr1GST-R primer set and cloning it into the PGEX-GST vector (a GST tag overexpression vector) to produce PGEX-GST-Vatr1. Meanwhile, the PGEX-His-Vatr1-GFP *vatr1* vector was produced by introducing the *vatr1* coding region as well as a 6×His sequence and the *gfp* gene, which were amplified using the vatr1-MST-F/vatr1-MST-R and GFP-MST-F/GFP-MST-R primer sets, respectively, into a PGEX vector lacking its GST tag (amplified using the PGEX-F/PGEX-R primer set). Both plasmids were transformed into *E. coli* BL21 (DE3) by heat shock for protein expression.

The compatibility of each predicted signal peptide with the Tat system was investigated using the MC4100Δ*ssamiAC* strain of *E. coli* and P*_tatABC_*-driven expression of a signal peptide-AmiA fusion proteins. The DNA sequences encoding the Cm signal peptide were prepared by PCR with the primer sets shown in Table S8, respectively, and cloned into a linearized pssAmiA-AmiAH vector from which the AmiA signal peptide sequence had been excised using the one-step cloning kit (TransGen Biotech). The resulting plasmids, respectively, were transformed into the MC4100Δ*ssamiAC* strain by electroporation.

All primers used during the cloning procedures were designed according to the instructions of the In-Fusion cloning system, which produces at least a 20 bp overlap region in the resulting PCR products.

### Sample preparation and RNA extraction

The bacterial samples used to identify changes in gene expression during the infection process were prepared using a method adapted from a previous study (65). The samples used to represent time 0 in the simulated infection process were produced by inoculating LB with fresh LBA colonies of the WT and the Δ*rel* mutant. After 24 h incubation at 28°C with shaking (150 rpm), the bacteria were harvested by centrifugation (8,000 rpm for 10 min) and washed in 0.85% NaCl, before being briefly resuspended in mM9 and re-harvested and snap-frozen. Meanwhile, an identical set of samples was resuspended in mM9 supplemented with 10 mL L^−1^ tomato xylem sap to mimic the rest of the infection process. In this case, the starting bacterial concentration was standardized to an OD_600_ of 0.04. The culture was incubated at 28°C with shaking (150 rpm) and a series of bacterial samples collected at different time points: 1, 3, 6, 12, 24, and 36 hpi and snap-frozen until required. Similar samples were also prepared for the RNA-seq analysis, but in this case, only the time 0 and 36 hpi samples were compared. Each sample was represented by three independent replicates per treatment.

The total RNA from each sample was extracted using the TransZol Up Plus RNA Kit (TransGen Biotech, Beijing, China). Briefly, the cells were resuspended in 100 µL of freshly prepared 1× Tris-EDTA (TE) buffer containing 50 mg mL^−1^ lysozyme before being incubated at 37°C for 30 min. The samples were then harvested by centrifugation (10,000 rpm for 2 min), the supernatant was removed, and the remaining pellet was treated according to the protocol of the manufacturer. The resulting RNA samples were eluted with 50 µL of nuclease-free water and frozen at −80°C until required. The concentration and purity of total RNA were determined using the NanoDrop2000 system (Thermo Fisher Scientific, Beijing, China), and the RNA integrity was verified by electrophoresis in 1% (wt/vol) agarose.

### RNA sequencing and data analysis

The total RNA from the WT and Δ*rel* samples was first treated to remove ribosomal RNA using the Ribo-Zero Magnetic kit (Bacteria) from EpiCentre Biotechnologies (Illumina, CA, USA) before 5 µg from each was used to construct strand-specific transcriptome libraries with the TruSeq Stranded mRNA Sample Prep Kit (Illumina, CA, USA). The resulting libraries were quantified and subjected to quality control using the Bioanalyzer 2100 system (Agilent Technologies, Beijing, China) before undergoing paired-end sequencing on the Illumina HiSeq platform, as well as conventional sequencing on the Illumina Hiseq 2500 platform with a target read length of 150 nucleotides, both of which were performed by Sangon Biotech (Shanghai, China). The raw data obtained were evaluated and filtered, and the validated data aligned to the NCPPB382 reference genome using Bowtie2 to generate the count mapping information (66). The abundance of gene transcripts was determined by calculating the normalized reads per kilobase per million mapped reads. The data from the replicate samples were combined so that the final expression analysis represented the average of all the replicate data. DEGs were identified using DESeq2 1.12.4 software, which screened for *Q*-values ≤0.05 and log2 fold changes ≥1.

The DEGs obtained were first annotated using the COG (http://www.ncbi.nlm.nih.gov), Gene Ontology (GO; https://amigo.geneontology.org/amigo), and KEGG (http://www.genome.jp/kegg/) databases, before KEGG and COG functional enrichment analyses were performed using clusterProfiler 3.0.5 software, with enrichment contingent on a corrected *P*-value (*Q*-value) <0.05. Meanwhile, GO enrichment was performed using topGO 2.24.0 software, with enrichment contingent on a *Q*-value <0.05. The DEGs involved in the pathogenicity and the metabolism of exopolysaccharides were then selected and screened using TBtools-ll (Toolbox for Biologists) v.2.067 software to produce a heat map of differential gene expression.

### qPCR and data analysis

The cDNA samples used for the qPCR analysis were prepared using 1 µg of RNA and the HiScript II 1st Strand cDNA Synthesis Kit (Vazyme, Nanjing, China) following the protocol of the manufacturer. The qPCR itself was performed using the primers listed in Table S8 and 20 μL reaction mixtures containing 10.0 µL ChamQ SYBR Color qPCR Master Mix (Vazyme), 2.0 µL cDNA template, 0.4 µL of each primer (1 × 10^−5^ M), and 7.2 µL sterile distilled water, which were processed using the Applied Biosystems 7500 Fast Real-Time PCR System (Life Technologies, USA) with the following program: 95°C for 30 s, followed by 40 cycles of 95°C for 5 s, 60°C for 30 s, and 72°C for 30 s. The resulting data were normalized using the *gyrB* and *bipA* reference genes as detailed in a previous study (67), and the relative expression of each target gene was calculated using the 2^-ΔΔCt^ method (68). To determine the effect of (p)ppGpp on the candidate genes, the relative expression from the Δ*rel* mutant was compared to that of the WT at different time points post-inoculation. Each sample was represented by three biological replicates per treatment, and the entire experiment was performed three times.

### Biofilm and extracellular enzyme assay

The biofilm produced by the WT, Δ*rel*, and Δ*rel::rel* strains was assessed using crystal violet staining. Briefly, liquid LB cultures of each strain were incubated to the logarithmic growth stage, before the bacterial concentration of each sample was adjusted to an OD_600_ of 1.0, and 100 µL samples were transferred to 24-well plates containing 900 µL of LB. After 7 day incubation at 28°C, the liquid medium was removed, and the plate was gently rinsed three times with distilled water before being placed in an 80°C oven for 20 min to fix the biofilm to the plate surface. The adhered biofilm was then stained with 0.1% crystal violet for 30 min and rinsed three times with distilled water, after which it was eluted by the addition of 1 mL 95% ethanol to each well for a period of 2 h, and the absorbance at OD_590_ was measured using a UV spectrophotometer (UV5500, METASH, Shanghai, China).

The amylase, endocellulase, and xylanase activity of the different Cm strains was determined according to the methods used in previous studies, but with slight modifications (8, 69–71). Briefly, fresh LB cultures were diluted to an OD_600_ of 0.3, and 2 µL was inoculated onto mM9 solid medium containing either 0.05% (wt/vol) soluble starch, 0.5% xylan (wt/vol), or 0.5% (wt/vol) carboxymethyl cellulose (CMC, Sigma-Aldrich, St Louis, MO, USA) for the amylase, xylanase, and endocellulase assays, respectively. The test plates were then incubated at 28℃ for 4 days before further treatment. In the case of the amylase assay, the plates were then stained with 20 mL I_2_/KI (53.12 g KI, 0.203 g solid I_2_ per 100 mL) for 10 min and washed with 20 mL of 70% ethanol. Strains capable of producing amylase were easily identifiable from the transparent hydrolysis halo surrounding them in contrast to the dark staining of the uninoculated media. The xylanase and endocellulase activity of the various Cm strains was assessed by adding 20 mL of 0.1% Congo red solution to the plates for a period of 30 min. After removing the staining solution, the plates were bleached twice with 1 M NaCl, which allowed colonies with cellulase or xylanase activity to be identified by the hydrolysis halos surrounding them in contrast to the red or orange backgrounds.

### Plant pathogenicity assay

The pathogenicity of the various Cm strains was assessed in tomato plants (*Solanum lycopersicum*, cv. Moneymaker) grown from seed in a greenhouse with a controlled photoperiod of 16 h of light and constant temperature of 25°C with watering when necessary. The plants were prick-inoculated at approximately 5 weeks when they had reached the four-leaf stage with Cm colonies of uniform size (1–2 mm) taken from LB plates and applied to the plant stem between the two cotyledons. Identical plants were inoculated with sterile water for the negative control. The plants were then returned to the greenhouse, and 1 cm stem sections were taken from the wound sites at various time points post-inoculation (0, 3, 6, 12, 24, 36, 72, and 169 h) to assess the bacterial load during the pre-colonization period. After disinfection with 1% NaClO solution for 3 min and washing twice in sterile water, the stem sections were frozen in liquid nitrogen and homogenized in pre-cooled mortars. The resulting samples were then resuspended in 1 mL 0.85% NaCl and used to prepare a 10-fold dilution series, which was plated on fresh LBA. The number of viable colonies was counted after 3 day incubation at 28°C. Similarly, the progress of the Cm infection within the tomato xylem was assessed over a 15 day period. In this case, 1 cm stem sections were taken from different locations of the tomato stem (the point of inoculation, the center of the stem, and the growing tip) at 5, 10, and 15 dpi, and the bacterial load was determined as described above. Meanwhile, the symptoms of the disease were assessed at 21 dpi, with the plant being photographed and the size of the lesion calculated using the ImageJ 1.54f system according to the protocol of the previous study (72, 73). Each treatment was represented by six individual plants, with the entire experiment conducted three times.

### Expression and purification of Vatr1 and His-tagged Vatr1 proteins assay

The expression vectors PGEX-GST-Vatr1 and PGEX-His-Vatr1-GFP were used to express GST- and His-tagged fusions of the Vatr1 protein in *E. coli* BL21 (DE3). A starter culture was initially prepared by inoculating LB containing 50 µg mL^−1^ kanamycin overnight at 37°C with shaking (200 rpm), before fresh LB (50 µg mL^−1^ kanamycin) cultures were inoculated at a ratio of 1:100. After a further 2 h of incubation at 37°C, when the OD_600_ was approximately 0.6 to 0.8, protein expression was induced by the addition of 1 × 10^−3^ M isopropyl-beta-D-thiogalactopyranoside. The induction cultures were then incubated at 28°C with shaking (150 rpm) for 4 h before the bacteria were harvested by centrifugation (8,000 *× g* for 10 min at 4°C). After washing twice with 1× phosphate-buffered saline (PBS; NaCl, 8 g L^−1^; KCl, 0.2 g L^−1^; Na_2_HPO_4_, 1.44 g L^−1^; KH_2_PO_4_, 0.24 g L^−1^; pH = 7.4), the samples were frozen at −80°C until required. The protein samples themselves were prepared when necessary, by resuspending the frozen bacteria in 20 mL lysis buffer (5 × 10^−2^ M Tris-HCl, 0.15 M NaCl, pH = 8.0) and processing them by ultrasonic fragmentation for 10 min in a mixture of ice and water (Scientz Biotechnology, 120 hz). The cell debris was removed by centrifugation (8,000 × *g* for 10 min at 4°C), and the supernatant lysates were processed using ProteinIso resin (TransGen Biotech, Beijing, China) or ProteinIso Ni-NTA resin (TransGen Biotech, Beijing, China) for the GST- and His-tagged proteins, respectively, according to the instructions of the manufacturer. The GST-tagged Vatr1 was eluted by the addition of 1 mL elution buffer (5 × 10^−2^ M Tris-HCl [pH 8.0], 10 × 10^−2^ M reduced glutathione), while the His-tagged Vatr1 was recovered by gradient elution with an imidazole buffer (0.25 M, and 0.4 M). The GST tag was cleaved using thrombin to produce tag-free Vatr1 for the next experiments. The purified proteins were then concentrated and analyzed by SDS-PAGE, and the protein concentration was determined using the Cauloblue method.

### Western blotting

The protein samples used in the western blotting were prepared from liquid mM9 cultures either in the absence or presence of tomato xylem sap (10 mL L^−1^), and inoculated with overnight LB cultures to an OD_600_ of approximately 0.3. After incubation with shaking for approximately 12 h until an OD_600_ of 0.9 had been reached, the cells were harvested by centrifugation (8,000 rpm for 10 min at 4°C) and washed twice in 1× PBS, and the total bacterial protein was extracted using the gram-positive Bacterial Protein Extraction Kit (Bestbio, Nanjing, China). Briefly, each sample was resuspended in 800 µL protein extraction buffer and manually ground for 4 min. The samples were then shaken and mixed for 1–2 min and placed in an ice bath for 2 min, a process that was repeated three times. After centrifugation (12,000 rpm for 5 min at 4°C), the resulting supernatant was collected as the total intracellular protein of Cm. Meanwhile, the culture supernatant obtained after the first centrifugation was treated with 200 µL of protease inhibitor (Biorigin, Beijing, China) and sterilized by filtration. The treated supernatant was then loaded into pre-conditioned dialysis bags and dialyzed overnight at 4°C against dialysis buffer (2 × 10^−2^ M Tris-HCl, 1 × 10^−2^ M NaCl, pH 7.4). The dialyzed solution was then concentrated by vacuum freeze-drying to obtain the final sample of extracellular proteins from Cm. The resulting proteins were separated using SDS-PAGE and transferred onto polyvinylidene fluoride (PVDF) membranes. After blocking with 5% skimmed milk powder, the membrane was incubated with a 1:1,000 dilution of anti-Flag mouse monoclonal antibodies at room temperature for 1 h and washed three times with TBST buffer (Tris 2 × 10^−2^ M, NaCl 0.3 M, Tween-20 0.08% [vol/vol]). The membranes were then incubated with a 1:5,000 dilution of goat anti-mouse antibodies at room temperature for 1 h and washed three times with TBST buffer before being visualized using the Azure Multifunctional Molecular Imaging System C600 (CA, USA).

### Electrophoretic mobility shift assay

The DNA substrates used for the DNA-binding activity analysis were obtained by amplifying the *xysB* promoter from WT genomic DNA with a fluorescein amidite (FAM)-labeled primer set (xysBProbeF/xysBProbeR). The resulting DNA probes were frozen at −20°C until required. Prior to electrophoresis, the probes were thawed and co-incubated with different concentrations (4–20 × 10^−6^ M) of Vatr1 test protein in EMSA buffer (5 × 10^−2^ M Tris-HCl, pH 7.5; 1 × 10^−2^ M MgCl_2_; 1 × 10^−3^ M DTT; and 5 × 10^−2^ M NaCl) at 25°C for 30 min. The 20 µL samples were then loaded on a 6% polyacrylamide-Tris-borate-EDTA gel and processed at 130 V for 1 h on ice. After electrophoresis, the samples were visualized using the Amersham ImageQuant 800 system (Cytiva, Japan).

### Microscale thermophoresis

Microscale thermophoresis was used to perform ligand binding assays to investigate the interactions between purified Vatr1, *xysB* promoter DNA, and ppGpp. All experiments were conducted using the Monolith NT.115 system (NanoTemper Technologies GmbH, Munich, Germany) with MO.Control v.1.6.1 software. The Red LED power was set at 50%–100% and infrared laser power to 75%. The purified Vatr1-GFP fusion protein was resuspended in MST buffer (2 × 10^−2^ M HEPES-Na, 2 × 10^−2^ M KCl, 2 × 10^−2^ M MgCl_2_, 0.2 M NaCl), either in the absence or presence of ppGpp (5 × 10^−3^ M), to a final concentration of 4 × 10^−2^ M, and titrated against the *xysB* promoter DNA at concentrations ranging from 0.75 to 3 × 10^−3^ M. The resulting fluorescence was measured at 680 nm and analyzed using MO.Affinity.Analysis v.2.3 software.

### Amidase reporter assay

The amidase reporter analysis utilizing the *E. coli* MC4100Δss*amiAC* strain was conducted according to the protocol described in a previous study (42). The chain-forming phenotypes and recovery of outer membrane integrity were assessed by microscopy (Zeiss LSM 800) and a 2% SDS sensitivity assay, respectively.

### Molecular docking

The 3D structures of the Vatr1 protein (A5CUE1), ppGpp (PubChem CID: 135402035), which were obtained from the AlphaFold Protein Structure Database and PubChem database, respectively, were imported into the Discovery Studio 2020 Client software (https://discover.3ds.com/discovery-studio-visualizer-download). The molecular docking was conducted using the CDOCKER semi-flexible docking method using the default parameters, and with only one -CDOCKER_ENERGY maximal conformation being retained. The resulting molecules were then visualized using Pymol software (https://pymol.org/2/).

## Data Availability

The RNA-Seq raw data of this study have been deposited in the Sequence Read Archive (SRA) database under accession number PRJNA1202210.

## SUPPLEMENTAL MATERIAL

Supplemental figures msystems.00135-25-s0001.pdf Fig. S1 to S6.

Table S1 msystems.00135-25-s0002.docx Summary of RNA-seq data for 12 samples induced in mM9 supplemented with 10 mL/L tomato xylem sap.

Table S2 msystems.00135-25-s0003.docx Database used in PCA.

Table S3 msystems.00135-25-s0004.docx Database used in Δrel-36 vs WT-36 COG enrichment analysis.

Table S4 msystems.00135-25-s0005.docx Database used in Δrel-36 vs WT-36 KEGG enrichment analysis.

Table S5 msystems.00135-25-s0006.docx TPM database used in heat map analysis of EPS.

Table S6 msystems.00135-25-s0007.docx TPM database used in heat map analysis of virulence genes.

Table S7 msystems.00135-25-s0008.docx Strains and plasmids used.

Table S8 msystems.00135-25-s0009.docx Primers used for the qPCR analysis.

ASM does not own the copyrights to Supplemental Material that may be linked to, or accessed through, an article. The authors have granted ASM a non-exclusive, world-wide license to publish the Supplemental Material files. Please contact the corresponding author directly for reuse.
