# Supplementary material for: Bacterial alarmone (p)ppGpp mediates the pathogenicity of Clavibacter michiganensis via a dual mechanism that affects both enzyme production and the Tat secretion system
Source: mSystems. 2025 Aug 4;10(9):e00135-25. doi: 10.1128/msystems.00135-25 (PMC12455917; doi:10.1128/msystems.00135-25)
Supplement: Supplemental figures — Fig. S1 to S6. [file msystems.00135-25-s0001.pdf]

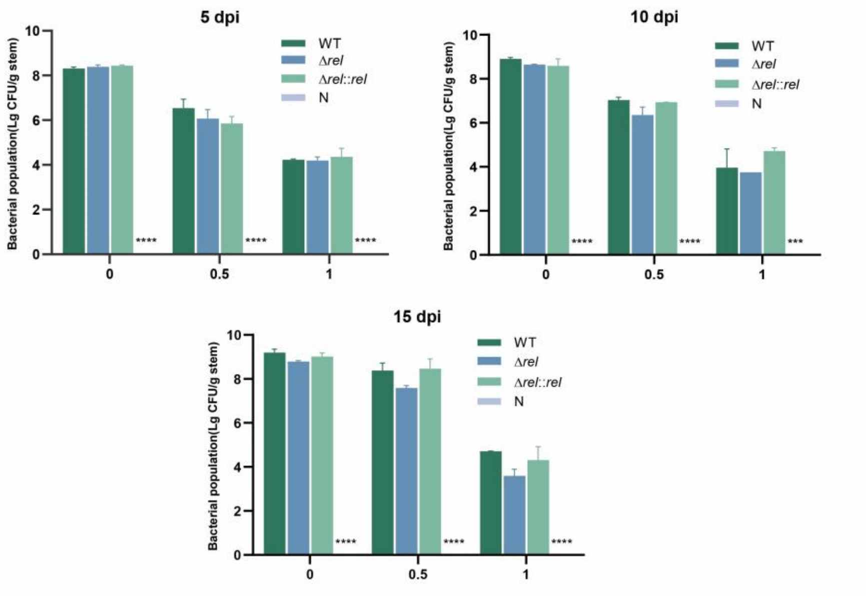

Figure S1. Effect of (p)ppGpp on bacterial titers of *Clavibacter michiganensis* from infected tomato stems. The tomato seedlings (*Solanum lycopersicum* 'Moneymaker') were prick inoculated at the four-leaf stage and bacterial titers assessed by reisolation from stem sections taken from various locations from the initial site of inoculation. Data represents the mean ± SD, while asterisks **** indicate significant differences ($p < 0.0001$) according to a two-way analysis of variance [ANOVA]).

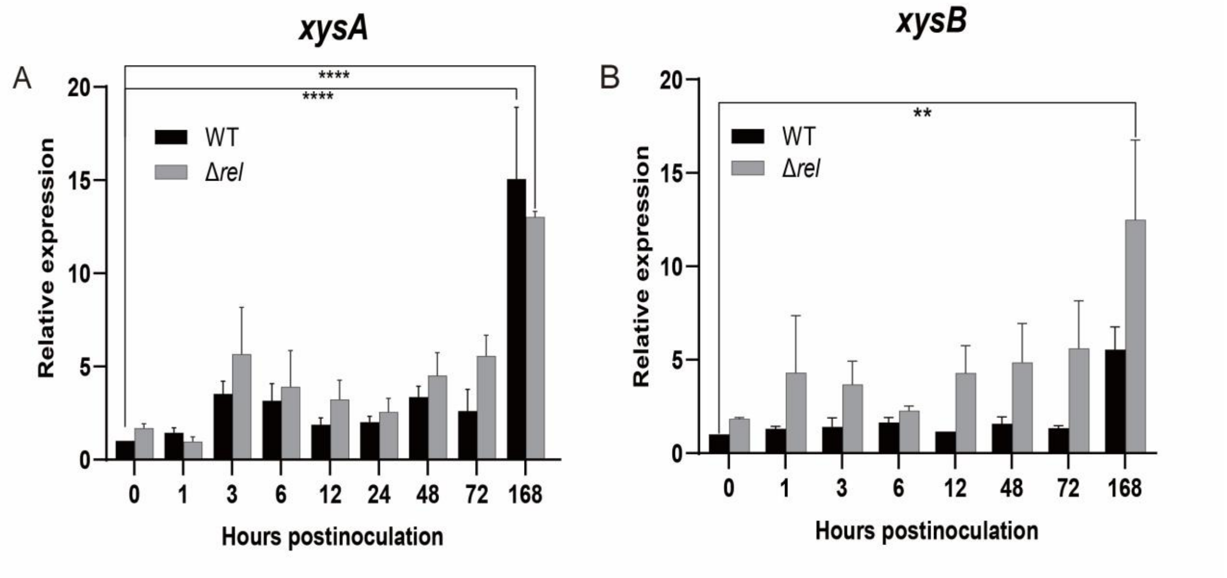

Figure S2. Relative expression levels of *xysA* (A) and *xysB* (B) in tomato stems at different time points post-inoculation with *Clavibacter michiganensis*. Data represents the mean ± SD, while asterisks **** indicate significant differences ($p < 0.0001$) according to a one-way ANOVA in comparison to the WT at time zero.

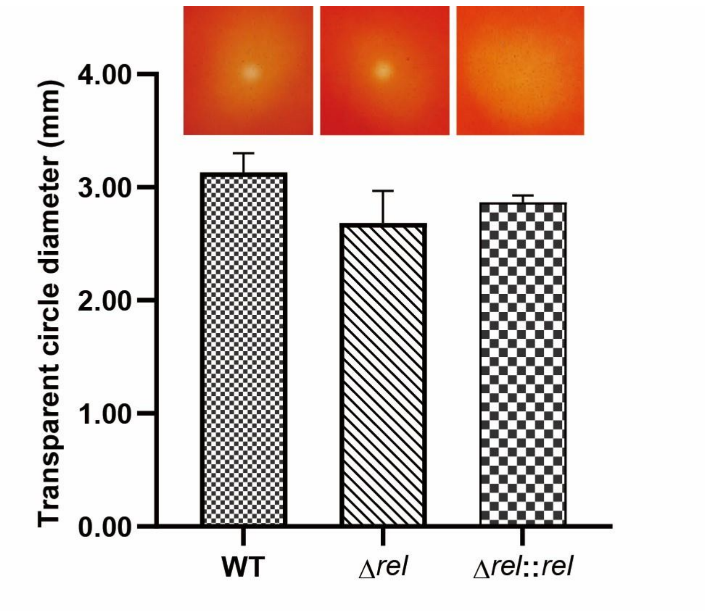

Figure S3. Hydrolysis halos associated with the cellulase activity of a *Clavibacter michiganensis* wild-type (WT) and (p)ppGpp-deficient mutant (Δ*rel*), with graph showing the corresponding halo measurements.

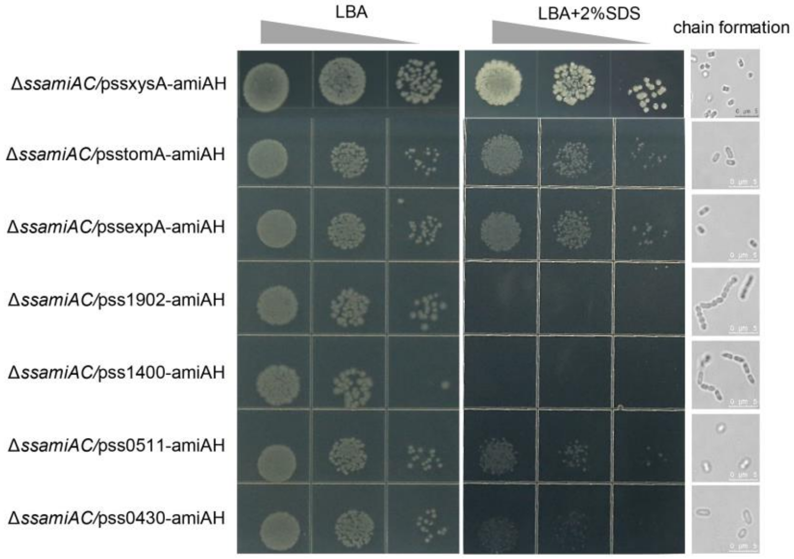

Figure S4. Verification of six Tat compatible signal peptides by amidase reporter assay. Six signal peptides were validated, including those from xylanases (XysA, TomA), putative expansin (ExpA), predicted amylase (CMM_0430, CMM_0511), and amine oxidase (CMM_1902), putative esterase (CMM_1400). The results confirmed that the signal peptides of XysA, TomA, ExpA, CMM_0430, and CMM_0511 are Tat system substrates. Successful complementation of the Δ*ssamiAC* phenotype, characterized by restored growth on LBA supplemented with 2% SDS and the absence of linked cell chains (observed via Zeiss LSM 800 microscope), verified their functionality. Gray sloping wedges indicate increasing inoculum dilutions (10^{-3}–10^{-5}), and scale bars represent 5 μm.

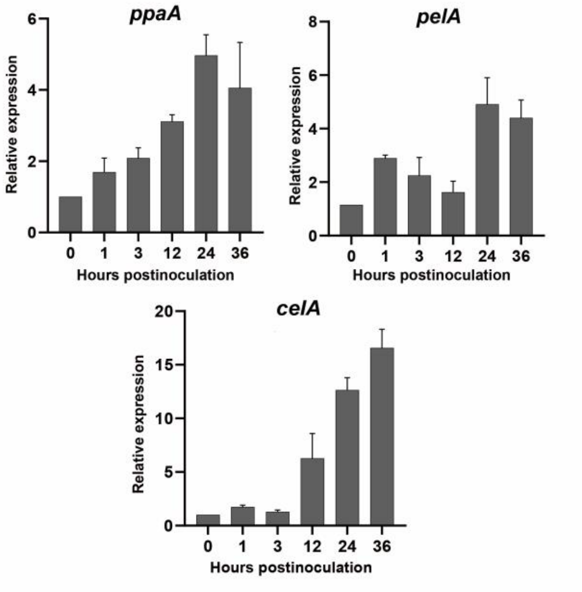

Figure S5. Expression levels of serine protease genes from *Clavibacter michiganensis* during a simulated infection (mM9 medium supplemented with 10 mL L^{-1} tomato xylem sap). Samples were taken at various time points (0, 1, 3, 12, 24, 36 hpi) and normalized by comparison to the *gyrB* and *bipA* reference genes, with their expression at time zero considered to be 1.

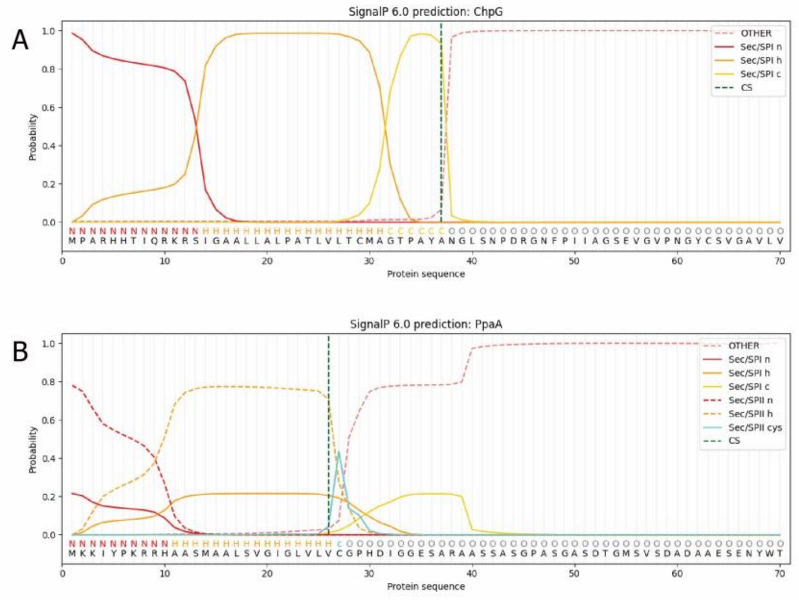

Figure S6. Predicted signal peptides of the cell wall-degrading enzymes ChpG and PpaA according to SignalP 6.0 software. (A) The ChpG signal peptide (Sec/SPII) had a likelihood of 0.9814, with cleavage site between residues 37 and 38. (B). Meanwhile, the PpaA lipoprotein signal peptide (Sec/SPII) had a likelihood of 0.7846 and cleavage site between residues 26 and 27.
