# Supplementary material for: Bacterial alarmone (p)ppGpp mediates the pathogenicity of Clavibacter michiganensis via a dual mechanism that affects both enzyme production and the Tat secretion system
Source: mSystems. 2025 Aug 4;10(9):e00135-25. doi: 10.1128/msystems.00135-25 (PMC12455917; doi:10.1128/msystems.00135-25)
Supplement: Table S1 — Summary of RNA-seq data for 12 samples induced in mM9 supplemented with 10 mL/L tomato xylem sap. [file msystems.00135-25-s0002.docx]

Table S1. Summary of RNA-seq data for 12 samples induced in mM9 supplemented with 10 mL/L tomato xylem sap.

| **Sample** | **Sample ID^a^** | **Raw reads** | **Clean Reads** | **Clean Bases (bp)** | **Q20 (%)^b^** | **Q30 (%)^c^** |  |
| --- | --- | --- | --- | --- | --- | --- | --- |
| WT 0 h | WT-0_1 | 21950492 | 21291146 | 3013117227 | 98.64 | 95.25 | |
|  | WT-0_2 | 18803726 | 18273338 | 2584383339 | 98.65 | 95.26 | |
|  | WT-0_3 | 18877448 | 18328664 | 2561500948 | 98.68 | 95.37 | |
| Δ*rel* 0 h | Δ*rel*-0_1 | 25889476 | 25081540 | 3584579370 | 98.57 | 95.04 | |
|  | Δ*rel*-0_2 | 19007188 | 18408182 | 2620642557 | 98.57 | 95.04 | |
|  | Δ*rel*-0_3 | 22282388 | 21679362 | 3066422521 | 98.66 | 95.32 | |
| WT 36 h | WT-36_1 | 22927784 | 22188012 | 3148260123 | 98.54 | 94.87 | |
|  | WT-36_2 | 19653352 | 19032586 | 2658156949 | 98.59 | 95.02 | |
|  | WT-36_3 | 21883662 | 21235500 | 2970974147 | 98.60 | 95.04 | |
| Δ*rel* 36 h | Δ*rel*-36_1 | 22126274 | 21332202 | 3010639577 | 98.49 | 94.83 | |
|  | Δ*rel*-36_2 | 20117522 | 19443954 | 2760218314 | 98.52 | 94.90 | |
|  | Δ*rel*-36_3 | 27442764 | 26396052 | 3784114098 | 98.40 | 94.55 | |

^a^ 1, 2 and 3 represent three independent biological replicates

^b^ Q20: The percentage of bases with a Phred value>20

^c^ Q30: The percentage of bases with a Phred value>30
