# Supplementary material for: Bacterial alarmone (p)ppGpp mediates the pathogenicity of Clavibacter michiganensis via a dual mechanism that affects both enzyme production and the Tat secretion system
Source: mSystems. 2025 Aug 4;10(9):e00135-25. doi: 10.1128/msystems.00135-25 (PMC12455917; doi:10.1128/msystems.00135-25)
Supplement: Table S2 — Database used in PCA. [file msystems.00135-25-s0003.docx]

Table S2. Database used in Principal Component Analysis (PCA).

|  | **Δ*rel*-0_1** | **Δ*rel*-0_2** | **Δ*rel*-0_3** | **Δ*rel*-36_1** | **Δ*rel*-36_2** | **Δ*rel*-36_3** | **WT-0_1** | **WT-0_2** | **WT-0_3** | **WT-36_1** | **WT-36_2** | **WT-36_3** |
| --- | --- | --- | --- | --- | --- | --- | --- | --- | --- | --- | --- | --- |
| PC1 | -42.5329 | -42.5817 | -40.8047 | 41.95288 | 44.95519 | 48.96447 | -42.0291 | -42.1497 | -41.2424 | 32.762 | 46.80077 | 35.90498 |
| PC2 | -9.74848 | -9.80347 | -10.668 | -29.5308 | -30.0369 | -29.2419 | 6.592821 | 7.520173 | 8.207574 | 34.50776 | 29.58332 | 32.6179 |
| PC3 | 19.22135 | 21.61414 | 21.93233 | -2.13728 | -5.37118 | -8.46455 | -21.89 | -22.8448 | -21.1274 | 11.8543 | -2.12734 | 9.340408 |
| PC4 | -7.22684 | -0.3888 | -1.10765 | 6.531248 | 2.305714 | -3.49209 | 4.859602 | 0.143936 | 0.159529 | 15.38078 | -22.6226 | 5.45713 |
| PC5 | -0.4249 | -0.00256 | 0.804708 | 0.853635 | -0.743 | -0.15514 | 0.604936 | -1.1042 | 0.232848 | 10.1748 | 3.917824 | -14.159 |
| PC6 | 3.796538 | -0.73216 | -2.25081 | -10.8138 | 1.318982 | 9.540577 | -1.66544 | 2.967331 | -1.92229 | 3.36131 | -3.61409 | 0.013844 |
| PC7 | -7.13569 | -0.06357 | 7.773488 | -4.07378 | 0.576801 | 2.738549 | 8.027771 | -4.02163 | -4.01435 | -1.3294 | 1.203463 | 0.318342 |
| PC8 | 1.583002 | -3.2337 | 1.676466 | 5.117323 | -10.8753 | 5.835296 | 0.825328 | 1.315426 | -2.14217 | 0.037521 | -0.7263 | 0.587073 |
| PC9 | 5.777453 | -0.8215 | -4.78668 | 1.070334 | 1.533523 | -2.72154 | 7.192021 | 0.457038 | -8.10462 | -0.09422 | 0.672789 | -0.17459 |
| PC10 | 1.324588 | -7.88607 | 6.408902 | 0.008457 | 2.928257 | -2.95337 | -2.76627 | 5.492331 | -2.65611 | 0.412214 | -0.17552 | -0.13741 |
| PC11 | -4.79507 | 5.689921 | -0.7593 | 0.683334 | -0.66607 | -0.11849 | -2.17281 | 7.030533 | -4.98889 | -0.26549 | 0.860907 | -0.49857 |
| PC12 | 6.78E-14 | 1.17E-14 | -6.63E-14 | 6.53E-14 | 1.76E-14 | -1.60E-14 | -8.84E-15 | -2.11E-14 | 3.20E-14 | -2.83E-14 | -2.64E-14 | 9.21E-15 |
