# Supplementary material for: Bacterial alarmone (p)ppGpp mediates the pathogenicity of Clavibacter michiganensis via a dual mechanism that affects both enzyme production and the Tat secretion system
Source: mSystems. 2025 Aug 4;10(9):e00135-25. doi: 10.1128/msystems.00135-25 (PMC12455917; doi:10.1128/msystems.00135-25)
Supplement: Table S3 — Database used in Δrel-36 vs WT-36 COG enrichment analysis. [file msystems.00135-25-s0004.docx]

Table S3. Database used in Δ*rel*-36 vs WT-36 COG enrichment analysis.

| **id** | **Description** | **Significant** | **Annotated** | **Pvalue** | **Qvalue** | **Signi_symbol** |
| --- | --- | --- | --- | --- | --- | --- |
| **Δ*rel*-36_vs_WT-36. up COG enrichment** | | | | | | |
| J | Translation, ribosomal structure and biogenesis | 63/205 | 153/1968 | 5.01E-26 | 8.44E-25 | infB(1.1896442783),rbfA(1.68567350626),rpsA(1.0608861655),rpsT(1.75148630864),rplK(1.58017254754),rplA(1.98258814934),rplJ(1.54540937009),rplL(1.00141746157),CMM_2644(1.52996883441),CMM_1851(1.02507532332),pthA(1.19439668428),tsfA(1.72411833234),rpsB(1.41586269197),rplQ(2.15659649977),pnpA(1.13547277182),rpsD(1.94310570705),CMM_2461(1.00941587366),rpsQ(2.80843502542),rpmC(2.5827696883),rpsH(2.18805863782),rplE(3.04576350725),rplX(2.87996001767),rplN(2.42881870543),rpmD(1.5532674892),rpsE(1.78014886067),rplR(2.4989853672),rplF(3.0024707633),rpmG(1.94294300439),rpmB(1.32503943821),rpsN(1.36944734964),rplO(1.68738046855),rpsJ(1.93672689149),rplC(1.79263290219),rplD(2.4531075209),rplW(2.7844270001),rplB(2.80097675184),rpsS(2.55591468849),rplV(2.65974304838),rpsC(2.69853275245),rplP(2.75820175895),rpmJ(2.49530542215),rpsM(1.88382070887),rpsK(2.09093277668),rplM(1.3017158205),rimM(1.98001062656),CMM_1691(1.00287654832),rpsG(1.71641873724),rpsL(1.70505581274),tufA(1.87235850189),fusA(2.30580770013),CMM_2487(1.28689052316),cysS1(1.20869537818),CMM_2008(1.37268038623),rplT(1.39279383481),pheS(1.04448125483),trmD(1.45025054531),rplS(1.62740605118),CMM_0155(1.51414750156),infC(1.306128479),rpmI(1.07820057768),rpsI(1.93190140394),rnpA(1.44470557533),rplU(1.3809217673) |
| G | Carbohydrate transport and metabolism | 49/205 | 250/1968 | 2.10E-06 | 1.77E-05 | abfA1(1.31732500242),CMM_0945(1.11154910061),CMM_0422(1.2618276345),fruK(1.01482926508),CMM_1820(1.67776602457),CMM_2437(2.16017702489),CMM_2699(2.4669921083),CMM_2698(2.57873468579),CMM_2438(1.08865761982),CMM_2239(2.78620633484),CMM_2238(1.98036257441),CMM_2781(1.38833609754),CMM_2783(1.89494872338),CMM_2782(2.05665925806),CMM_2240(2.20068788596),abfA2(1.0653654283),CMM_0108(1.7957476584),bglG(1.48159166189),bglF(1.44849708819),CMM_0879(1.24438321319),araA(1.38054493029),araD(1.48091496611),CMM_2411(1.03816641327),CMM_2412(1.18417048926),CMM_0270(1.3264530177),CMM_1262(2.25641497786),CMM_1261(1.16594861454),xysB(1.12205869018),xysA(1.44553958768),celB(1.23481368862),bgaB(1.28122412431),CMM_1286(1.50050943873),CMM_1243(1.17413075177),CMM_1244(4.04819068745),CMM_2697(3.27549756545),CMM_0296(2.02567492201),wzt(1.17036249169),CMM_0359(1.56203336415),CMM_0196(1.45277244516),CMM_0197(1.93621549163),CMM_0880(1.71091844248),CMM_0084(1.56934734034),bglB(1.63702635653),bglC(1.52129687422),CMM_0110(1.83915450072),CMM_0360(1.34687139303),bglE(1.37211734419),bglD(1.25249865978),CMM_2578(1.25550784453) |
| P | Inorganic ion transport and metabolism | 16/205 | 135/1968 | 0.327512 | 0.935665 | CMM_1810(1.25739727141),CMM_2939(1.07718193783),CMM_2190(1.33555251117),nhaA1(1.04727590118),pstB(2.03688506812),cynT(1.04629008029),CMM_2282(1.11477298021),pitB(1.05991399864),trkG(1.22721038391),CMM_2283(1.3235595515),CMM_2051(1.39170176969),sulP(2.44638409257),fecE(1.15887790863),cynX(1.12677508231),CMM_1116(1.15385544358),CMM_1570(1.0242539603) |
| U | Intracellular trafficking, secretion, and vesicular transport | 3/205 | 22/1968 | 0.406182 | 0.935665 | secD(1.21829784219),lepB(1.86960577851),CMM_2975(2.09276850643) |
| I | Lipid transport and metabolism | 7/205 | 71/1968 | 0.621747 | 0.935665 | tagD(2.63735662598),fadA(1.68241721123),fadB(1.35836771837),gcdH(1.85702958362),CMM_0781(1.00767955201),CMM_2155(1.17748499276),CMM_2792(1.0834584025) |
| D | Cell cycle control, cell division, chromosome partitioning | 2/205 | 24/1968 | 0.73132 | 0.935665 | CMM_2670(1.45330026587),parA(1.8573401104) |
| M | Cell wall/membrane/envelope biogenesis | 11/205 | 131/1968 | 0.823218 | 0.935665 | lepA(1.05899722804),ftsI(1.56708045579),mraW(1.24621487955),tagD(2.63735662598),CMM_2079(1.10988411503),wcnC(1.12459780344),wcoF(1.21400606991),CMM_1286(1.50050943873),CMM_2674(1.09174308256),wzt(1.17036249169),CMM_2798(1.26873289267) |
| C | Energy production and conversion | 9/205 | 119/1968 | 0.890197 | 0.935665 | CMM_0321(1.240329207),araB(1.32508377844),CMM_0530(2.21575396631),atpG(2.13208886135),atpD(2.02740700552),atpH(1.03216159702),atpA(2.0594197777),cytB(1.26362260427),atpC(1.56021529829) |
| L | Replication, recombination and repair | 7/205 | 102/1968 | 0.922063 | 0.935665 | dnaA(2.4308145295),CMM_2644(1.52996883441),pknD(1.29727683066),CMM_1805(2.35430289011),hupB(1.2522468053),CMM_2619(2.42025505482),rnhB(1.10225217242) |
| V | Defense mechanisms | 1/205 | 29/1968 | 0.959815 | 0.935665 | CMM_0869(1.15091341572) |
| K | Transcription | 14/205 | 196/1968 | 0.961248 | 0.935665 | nusG(1.74898538688),rncA(1.3425711292),CMM_2644(1.52996883441),pknD(1.29727683066),cspA2(1.16604040512),CMM_1063(1.21087228075),CMM_0295(1.53681359565),rpoA(2.48229272995),CMM_1978(1.03248104346),catR(1.55652431807),rpoB(1.84851990332),rpoC(1.66928919144),CMM_0560(1.07482153886),parB1(2.01447166909) |
| F | Nucleotide transport and metabolism | 3/205 | 66/1968 | 0.975262 | 0.935665 | CMM_1820(1.67776602457),purN(1.16466840635),cmkA(1.49280045007) |
| H | Coenzyme transport and metabolism | 4/205 | 87/1968 | 0.985685 | 0.935665 | ilvB(1.45377573342),ilvC(1.7277970941),fecE(1.15887790863),hemN(1.17531459693) |
| S | Function unknown | 6/205 | 140/1968 | 0.998045 | 0.935665 | CMM_1425(1.27559171319),CMM_0870(1.15307827233),CMM_2454(1.79013041231),CMM_0156(1.42251856108),CMM_0520(1.8714396275),CMM_2976(1.30567081637) |
| R | General function prediction only | 14/205 | 248/1968 | 0.998313 | 0.935665 | obgA(1.7875344638),CMM_2567(1.0677377319),CMM_1820(1.67776602457),CMM_1324(1.40744629602),CMM_0409(1.16121602642),pknD(1.29727683066),CMM_1892(1.26805431856),CMM_2052(1.00169702424),CMM_0172(1.22723075234),CMM_1368(1.39764862395),CMM_1726(1.1290906816),CMM_0081(1.69630901849),CMM_0417(1.54777910172),CMM_2974(2.1302182291) |
| O | Posttranslational modification, protein turnover, chaperones | 1/205 | 65/1968 | 0.999308 | 0.935665 | CMM_0133(1.04817899773) |
| T | Signal transduction mechanisms | 2/205 | 96/1968 | 0.999749 | 0.935665 | pknD(1.29727683066),CMM_1568(1.05726733401) |
| E | Amino acid transport and metabolism | 6/205 | 201/1968 | 0.999992 | 0.935665 | ilvB(1.45377573342),ilvH(1.38644151519),ilvC(1.7277970941),proB(1.08361000488),CMM_2683(1.27942132593),hisF(1.01287256854) |
| **Δ*rel*-36_vs_WT-36. down COG enrichment** | | | | | | |
| C | Energy production and conversion | 39/256 | 119/1968 | 5.87E-09 | 9.88E-08 | CMM_1956(-1.184720406),sucA(-1.32820690554),mnhAB(-1.51460261621),mnhD(-3.72832486535),CMM_2432(-1.71807194677),CMM_2662(-2.63467048131),CMM_1526(-2.60156495277),CMM_0211(-2.95327003799),CMM_0914(-1.37921491714),icdA(-1.10612878991),ctaC(-1.28353164663),ctaD(-1.41118285929),CMM_1274(-2.3227034571),CMM_1879(-1.11802535836),CMM_0495(-1.68406681082),cydB(-1.23731775318),cydA(-1.52101884794),CMM_1326(-3.23504709512),CMM_0735(-1.11411010998),CMM_2278(-3.40938504753),qorA(-1.82040747109),CMM_1929(-3.67055132933),CMM_1925(-1.96344714877),CMM_0396(-1.68074177322),CMM_2778(-3.37551043634),acnA(-1.64905316715),glpD(-3.27378473922),putA(-1.23471238851),CMM_2483(-2.29479280613),CMM_0970(-1.66032086286),sdhD(-2.12242978614),sdhC(-2.35430438003),glpK(-1.62888990646),sdhB(-1.61744251486),ackA(-1.07977907398),qcrA(-1.29136831202),qcrB(-1.83868208175),ctaE(-1.31260964599),CMM_2796(-2.02219487564) |
| O | Posttranslational modification, protein turnover, chaperones | 17/256 | 65/1968 | 0.002794 | 0.023532 | trxA(-1.67716035652),trxB1(-2.44975356934),clpC(-1.30936644067),CMM_2759(-1.0967295527),sbtB(-1.43077850371),CMM_1731(-2.26333957962),CMM_1824(-1.326640512),ppiA(-1.27318525104),CMM_0709(-1.49042222565),hsp20(-1.36440171589),CMM_2111(-1.3932644919),msrA(-4.50816252683),CMM_2482(-3.72189242249),resC(-2.32909736366),trxC(-3.78711530441),dnaK(-1.18851260309),CMM_2121(-2.25504097028) |
| D | Cell cycle control, cell division, chromosome partitioning | 6/256 | 24/1968 | 0.080918 | 0.454275 | ftsX(-1.67733165004),ftsZ(-2.18447791732),ftsW2(-1.39744548903),pCM2_0066(-1.73203373353),ftsK(-1.4993342929),parA(-2.88105847003) |
| S | Function unknown | 23/256 | 140/1968 | 0.132861 | 0.559414 | CMM_1954(-2.11304541135),CMM_2225(-1.5056496245),CMM_1082(-2.43072273445),CMM_1854(-1.56176321213),CMM_1843(-2.77041578789),CMM_1613(-1.16344548984),CMM_0479(-3.36911394823),CMM_0671(-2.84287669891),CMM_0497(-1.84192492346),CMM_0640(-2.39765816501),CMM_0022(-1.41758541738),CMM_1514(-5.5743189749),CMM_0388(-2.30452077116),CMM_0394(-1.83591350526),CMM_1265(-1.73163562707),CMM_0144(-2.49245866095),CMM_1955(-1.92607470793),CMM_0602(-1.37280520112),CMM_2638(-2.20042067769),CMM_0343(-4.34624346342),CMM_1582(-1.58610653214),CMM_2129(-3.23419662591),CMM_2127(-1.28075605204) |
| L | Replication, recombination and repair | 16/256 | 102/1968 | 0.244208 | 0.822595 | CMM_2224(-1.3024063252),pin(-2.46554452366),dinB(-1.82825055226),CMM_2539(-1.12874056631),uvrD2(-1.15266690943),recQ2(-2.02549168183),recR(-1.81504566785),CMM_0900(-1.87984866308),CMM_2068(-1.34018303165),uvrC(-1.12300095905),CMM_2072(-1.83677409204),uvrB(-1.70001228157),uvrA(-2.43748339034),recA(-2.29940672189),CMM_1225(-1.38646680442),CMM_0574(-2.66339263046) |
| P | Inorganic ion transport and metabolism | 19/256 | 135/1968 | 0.391554 | 0.897707 | CMM_0426(-2.18506019501),mnhE(-3.50176856519),mnhF(-3.68680818798),mnhG(-3.01556840822),mnhAB(-1.51460261621),mnhD(-3.72832486535),mnhC(-2.52171260593),CMM_2768(-4.0622527353),CMM_1274(-2.3227034571),dpsB(-2.20772970098),dpsA(-3.96938340886),amtB(-4.74693868537),sseA(-1.0321032559),CMM_0396(-1.68074177322),CMM_1729(-1.05462562869),sodA(-2.16173747903),CMM_2102(-1.93679974197),copP(-1.05382212112),CMM_1588(-1.64385618977) |
| E | Amino acid transport and metabolism | 27/256 | 201/1968 | 0.460221 | 0.897707 | livM(-2.11909467077),livK(-3.77368075815),CMM_2969(-1.126676141),CMM_2212(-3.9872095616),gabP(-3.42945063302),CMM_1146(-1.60770462139),CMM_2185(-1.31674757012),glnA2(-1.47682018827),glnA1(-3.16248513795),CMM_2251(-1.77856861986),serC(-1.20082382902),dcpA(-1.62201307118),aldA(-3.18812553504),dapX(-1.73504135908),cysK(-1.08495641773),CMM_2873(-1.50348064185),ansP(-1.22462725261),CMM_1232(-4.22596008687),CMM_1588(-1.64385618977),CMM_1587(-2.95899610466),gluB(-1.51112317016),gluA(-2.33648028062),gluC(-1.73135483651),CMM_0888(-1.36932744556),CMM_1888(-2.20102621873),CMM_0563(-1.23476615781),glyA(-2.0251275891) |
| G | Carbohydrate transport and metabolism | 33/256 | 250/1968 | 0.494213 | 0.897707 | CMM_1145(-4.73359642594),CMM_2516(-1.81874389378),CMM_2512(-1.79399560005),CMM_1754(-1.4531938013),fbaA(-1.50085552655),glpF(-1.9065965798),CMM_1084(-1.06310755404),CMM_0201(-1.39769681264),galE2(-1.13489310677),CMM_2692(-3.93800598045),CMM_0495(-1.68406681082),glpX(-2.06150526611),enoA(-1.77645707446),gndA2(-1.28895847276),treZ(-1.168864493),glgC(-1.59548303979),glgB(-2.34778213262),CMM_2844(-1.04668768979),CMM_2842(-1.46290421332),CMM_2112(-2.02303148305),mtlA(-1.04276299708),gapA(-1.03928382314),talA(-1.26256397458),tktA(-1.55025652579),pgiA(-1.06514344716),manB(-1.2948869035),CMM_2485(-2.74261142561),CMM_1714(-2.70437261819),xylA(-1.11863448442),CMM_2316(-1.89757230906),bglA(-1.03780782635),CMM_2839(-1.9713489093),CMM_2831(-1.23039255297) |
| F | Nucleotide transport and metabolism | 9/256 | 66/1968 | 0.495302 | 0.897707 | nrdB(-1.33067102155),nrdA(-1.66936254733),CMM_1126(-1.92211182135),punA(-1.241791813),pyrG(-1.44328938267),CMM_2826(-3.55978245122),CMM_2822(-1.91639871585),pyrB(-1.74313463896),pyrR(-1.16841556678) |
| V | Defense mechanisms | 4/256 | 29/1968 | 0.533014 | 0.897707 | CMM_2903(-1.68408523111),CMM_0684(-2.92204485703),CMM_1240(-2.41077480964),CMM_1239(-2.45186988283) |
| Q | Secondary metabolites biosynthesis, transport and catabolism | 6/256 | 54/1968 | 0.723839 | 0.935669 | CMM_0680(-1.82437980169),fabB(-2.07852739734),CMM_0073(-1.42637621567),CMM_0469(-1.99287053594),CMM_2075(-1.4538899854),CMM_2825(-2.25864185015) |
| R | General function prediction only | 29/256 | 248/1968 | 0.773744 | 0.935669 | CMM_1706(-2.72420039009),CMM_2366(-2.93344880709),CMM_2694(-1.82168542072),CMM_0687(-1.6326686806),CMM_0064(-1.03987869688),CMM_2340(-3.76681234364),CMM_2914(-1.11213610954),CMM_0469(-1.99287053594),cpdA(-1.62897028417),CMM_2082(-1.21433327113),CMM_2060(-1.29496650936),wcmI(-1.8648910629),CMM_2856(-1.33262119275),qorA(-1.82040747109),CMM_1929(-3.67055132933),CMM_2045(-1.99663455436),CMM_0397(-1.32774435545),CMM_2778(-3.37551043634),CMM_1231(-2.62207718454),CMM_1729(-1.05462562869),CMM_1747(-2.15885978176),CMM_2028(-1.21107723311),CMM_1135(-3.69677618362),CMM_2102(-1.93679974197),CMM_2038(-1.04583472327),CMM_0976(-2.30027641572),CMM_0795(-1.81773754206),CMM_0562(-1.08710302415),CMM_2200(-1.09833442865) |
| T | Signal transduction mechanisms | 10/256 | 96/1968 | 0.822682 | 0.935669 | CMM_1331(-2.00281970238),CMM_2235(-2.94940459153),CMM_2655(-1.80688394049),kdpD(-1.31925196503),kdpE(-2.76284422599),CMM_2475(-1.09619285093),CMM_2277(-1.23448066213),relA(-19.8687622805),CMM_1136(-1.84741102597),gluB(-1.51112317016) |
| M | Cell wall/membrane/envelope biogenesis | 14/256 | 131/1968 | 0.828921 | 0.935669 | CMM_0685(-2.06482200106),galE2(-1.13489310677),CMM_1523(-1.44135456195),pbpC(-2.77684221514),murC(-1.0967608706),wcmN(-1.32988668183),wcmH(-1.61038769584),gmdA(-2.99451192704),CMM_1288(-2.53988465702),dapX(-1.73504135908),wcmF(-1.43769700349),CMM_2839(-1.9713489093),CMM_2831(-1.23039255297),ddlB(-2.96366101973) |
| K | Transcription | 20/256 | 196/1968 | 0.913497 | 0.935669 | sigH(-3.17467251504),CMM_2969(-1.126676141),sigK(-2.4212346268),CMM_1331(-2.00281970238),CMM_2867(-3.09549944924),CMM_2784(-1.46286619888),CMM_2655(-1.80688394049),CMM_0267(-3.34661206167),CMM_2525(-1.72057048435),cspA1(-2.60157475991),CMM_0674(-2.00289468451),sigA(-1.2729145585),kdpE(-2.76284422599),CMM_2475(-1.09619285093),relA(-19.8687622805),CMM_1136(-1.84741102597),CMM_1225(-1.38646680442),CMM_0344(-4.37662794908),CMM_0893(-2.11711071277),sigB(-2.35894246091) |
| I | Lipid transport and metabolism | 6/256 | 71/1968 | 0.91724 | 0.935669 | fabB(-2.07852739734),CMM_0073(-1.42637621567),CMM_0469(-1.99287053594),CMM_0268(-2.10145588991),wcnB(-1.71078221308),CMM_1945(-1.35517911942) |
| H | Coenzyme transport and metabolism | 4/256 | 87/1968 | 0.997915 | 0.935669 | CMM_1524(-1.72679008892),folD(-1.23852842948),serC(-1.20082382902),CMM_2909(-1.382626364) |
| J | Translation, ribosomal structure and biogenesis | 5/256 | 153/1968 | 0.999996 | 0.935669 | CMM_1146(-1.60770462139),CMM_1053(-4.10861695573),CMM_0679(-4.86536808265),CMM_1225(-1.38646680442),CMM_2824(-1.09777953522) |
