# Supplementary material for: Bacterial alarmone (p)ppGpp mediates the pathogenicity of Clavibacter michiganensis via a dual mechanism that affects both enzyme production and the Tat secretion system
Source: mSystems. 2025 Aug 4;10(9):e00135-25. doi: 10.1128/msystems.00135-25 (PMC12455917; doi:10.1128/msystems.00135-25)
Supplement: Table S4 — Database used in Δrel-36 vs WT-36 KEGG enrichment analysis. [file msystems.00135-25-s0005.docx]

Table S4. Database used in Δ*rel*-36 vs WT-36 KEGG enrichment analysis.

| **id** | **Description** | **Significant** | | **Annotated** | | **Pvalue** | **Qvalue** | | **Signi_symbol** | |  |  |
| --- | --- | --- | --- | --- | --- | --- | --- | --- | --- | --- | --- | --- |
| **Δ*rel*-36_vs_WT-36. up KEGG enrichment** | | | | | | | | | | | |  |
| ko03010 | Ribosome | | 44/109 | | 54/829 | 2.20E-33 | | 9.71E-32 | | rpsA(1.0608861655),rpsT(1.75148630864),rplK(1.58017254754),rplA(1.98258814934),rplJ(1.54540937009),rplL(1.00141746157),rpsB(1.41586269197),rplQ(2.15659649977),rpsD(1.94310570705),rpsQ(2.80843502542),rpmC(2.5827696883),rpsH(2.18805863782),rplE(3.04576350725),rplX(2.87996001767),rplN(2.42881870543),rpmD(1.5532674892),rpsE(1.78014886067),rplR(2.4989853672),rplF(3.0024707633),rpmG(1.94294300439),rpmB(1.32503943821),rpsN(1.36944734964),rplO(1.68738046855),rpmF(1.00233954938),rpsJ(1.93672689149),rplC(1.79263290219),rplD(2.4531075209),rplW(2.7844270001),rplB(2.80097675184),rpsS(2.55591468849),rplV(2.65974304838),rpsC(2.69853275245),rplP(2.75820175895),rpmJ(2.49530542215),rpsM(1.88382070887),rpsK(2.09093277668),rplM(1.3017158205),rpsG(1.71641873724),rpsL(1.70505581274),rplT(1.39279383481),rplS(1.62740605118),rpmI(1.07820057768),rpsI(1.93190140394),rplU(1.3809217673) | |  |
| ko00195 | Photosynthesis | | 5/109 | | 8/829 | 0.001455 | | 0.032171 | | atpG(2.13208886135),atpD(2.02740700552),atpH(1.03216159702),atpA(2.0594197777),atpC(1.56021529829) | |  |
| ko03020 | RNA polymerase | | 3/109 | | 4/829 | 0.008021 | | 0.118211 | | rpoA(2.48229272995),rpoB(1.84851990332),rpoC(1.66928919144) | |  |
| ko00940 | Phenylpropanoid biosynthesis | | 4/109 | | 8/829 | 0.013006 | | 0.143754 | | bglG(1.48159166189),bglF(1.44849708819),bglB(1.63702635653),bglC(1.52129687422) | |  |
| ko00460 | Cyanoamino acid metabolism | | 4/109 | | 11/829 | 0.044733 | | 0.395536 | | bglG(1.48159166189),bglF(1.44849708819),bglB(1.63702635653),bglC(1.52129687422) | |  |
| ko02010 | ABC transporters | | 18/109 | | 100/829 | 0.088159 | | 0.581296 | | CMM_2190(1.33555251117),pstB(2.03688506812),CMM_2239(2.78620633484),CMM_2238(1.98036257441),CMM_2781(1.38833609754),CMM_2783(1.89494872338),CMM_2782(2.05665925806),CMM_2240(2.20068788596),CMM_2282(1.11477298021),CMM_0879(1.24438321319),CMM_2283(1.3235595515),CMM_1243(1.17413075177),CMM_1244(4.04819068745),CMM_0296(2.02567492201),fecE(1.15887790863),CMM_0880(1.71091844248),CMM_0084(1.56934734034),CMM_0086(1.10152594121) | |  |
| ko00362 | Benzoate degradation | | 2/109 | | 5/829 | 0.131248 | | 0.581296 | | fadA(1.68241721123),gcdH(1.85702958362) | |  |
| ko00281 | Geraniol degradation | | 1/109 | | 1/829 | 0.131484 | | 0.581296 | | fadA(1.68241721123) | |  |
| ko00510 | N-Glycan biosynthesis | | 1/109 | | 1/829 | 0.131484 | | 0.581296 | | CMM_2079(1.10988411503) | |  |
| ko00642 | Ethylbenzene degradation | | 1/109 | | 1/829 | 0.131484 | | 0.581296 | | fadA(1.68241721123) | |  |
| ko00290 | Valine, leucine and isoleucine biosynthesis | | 3/109 | | 11/829 | 0.166146 | | 0.667765 | | ilvB(1.45377573342),ilvH(1.38644151519),ilvC(1.7277970941) | |  |
| ko00190 | Oxidative phosphorylation | | 5/109 | | 24/829 | 0.197723 | | 0.679232 | | atpG(2.13208886135),atpD(2.02740700552),atpH(1.03216159702),atpA(2.0594197777),atpC(1.56021529829) | |  |
| ko00500 | Starch and sucrose metabolism | | 6/109 | | 32/829 | 0.235169 | | 0.679232 | | bglG(1.48159166189),bglF(1.44849708819),celB(1.23481368862),sacB(1.16700565375),bglB(1.63702635653),bglC(1.52129687422) | |  |
| ko00332 | Carbapenem biosynthesis | | 1/109 | | 2/829 | 0.245817 | | 0.679232 | | proB(1.08361000488) | |  |
| ko00592 | alpha-Linolenic acid metabolism | | 1/109 | | 2/829 | 0.245817 | | 0.679232 | | fadA(1.68241721123) | |  |
| ko03008 | Ribosome biogenesis in eukaryotes | | 1/109 | | 2/829 | 0.245817 | | 0.679232 | | rncA(1.3425711292) | |  |
| ko00660 | C5-Branched dibasic acid metabolism | | 2/109 | | 8/829 | 0.283975 | | 0.73851 | | ilvB(1.45377573342),ilvH(1.38644151519) | |  |
| ko00071 | Fatty acid degradation | | 2/109 | | 9/829 | 0.335795 | | 0.823594 | | fadA(1.68241721123),gcdH(1.85702958362) | |  |
| ko00040 | Pentose and glucuronate interconversions | | 2/109 | | 10/829 | 0.386485 | | 0.823594 | | araA(1.38054493029),araB(1.32508377844) | |  |
| ko00770 | Pantothenate and CoA biosynthesis | | 3/109 | | 17/829 | 0.391207 | | 0.823594 | | ilvB(1.45377573342),ilvH(1.38644151519),ilvC(1.7277970941) | |  |
| ko03060 | Protein export | | 3/109 | | 17/829 | 0.391207 | | 0.823594 | | secD(1.21829784219),lepB(1.86960577851),CMM_2975(2.09276850643) | |  |
| ko00650 | Butanoate metabolism | | 2/109 | | 11/829 | 0.435449 | | 0.85532 | | ilvB(1.45377573342),ilvH(1.38644151519) | |  |
| ko00310 | Lysine degradation | | 1/109 | | 5/829 | 0.50672 | | 0.85532 | | gcdH(1.85702958362) | |  |
| ko00380 | Tryptophan metabolism | | 1/109 | | 5/829 | 0.50672 | | 0.85532 | | gcdH(1.85702958362) | |  |
| ko00910 | Nitrogen metabolism | | 1/109 | | 5/829 | 0.50672 | | 0.85532 | | cynT(1.04629008029) | |  |
| ko03018 | RNA degradation | | 2/109 | | 14/829 | 0.568423 | | 0.85532 | | pnpA(1.13547277182),CMM_0172(1.22723075234) | |  |
| ko03070 | Bacterial secretion system | | 2/109 | | 14/829 | 0.568423 | | 0.85532 | | secD(1.21829784219),CMM_2975(2.09276850643) | |  |
| ko01210 | 2-Oxocarboxylic acid metabolism | | 3/109 | | 22/829 | 0.569538 | | 0.85532 | | ilvB(1.45377573342),ilvH(1.38644151519),ilvC(1.7277970941) | |  |
| ko00053 | Ascorbate and aldarate metabolism | | 1/109 | | 6/829 | 0.571972 | | 0.85532 | | araD(1.48091496611) | |  |
| ko00520 | Amino sugar and nucleotide sugar metabolism | | 4/109 | | 31/829 | 0.599559 | | 0.85532 | | abfA1(1.31732500242),abfA2(1.0653654283),CMM_0110(1.83915450072),CMM_2578(1.25550784453) | |  |
| ko00240 | Pyrimidine metabolism | | 5/109 | | 39/829 | 0.599742 | | 0.85532 | | pnpA(1.13547277182),rpoA(2.48229272995),cmkA(1.49280045007),rpoB(1.84851990332),rpoC(1.66928919144) | |  |
| ko00230 | Purine metabolism | | 6/109 | | 49/829 | 0.643975 | | 0.889703 | | CMM_1820(1.67776602457),purN(1.16466840635),pnpA(1.13547277182),rpoA(2.48229272995),rpoB(1.84851990332),rpoC(1.66928919144) | |  |
| ko00670 | One carbon pool by folate | | 1/109 | | 9/829 | 0.720665 | | 0.895581 | | purN(1.16466840635) | |  |
| ko04112 | Cell cycle - Caulobacter | | 1/109 | | 9/829 | 0.720665 | | 0.895581 | | dnaA(2.4308145295) | |  |
| ko02020 | Two-component system | | 2/109 | | 19/829 | 0.737209 | | 0.895581 | | dnaA(2.4308145295),sacB(1.16700565375) | |  |
| ko00330 | Arginine and proline metabolism | | 1/109 | | 10/829 | 0.757796 | | 0.895581 | | proB(1.08361000488) | |  |
| ko00564 | Glycerophospholipid metabolism | | 1/109 | | 10/829 | 0.757796 | | 0.895581 | | tagD(2.63735662598) | |  |
| ko00280 | Valine, leucine and isoleucine degradation | | 1/109 | | 11/829 | 0.790031 | | 0.895581 | | fadA(1.68241721123) | |  |
| ko00340 | Histidine metabolism | | 1/109 | | 11/829 | 0.790031 | | 0.895581 | | hisF(1.01287256854) | |  |
| ko01212 | Fatty acid metabolism | | 1/109 | | 12/829 | 0.81801 | | 0.904116 | | fadA(1.68241721123) | |  |
| ko00970 | Aminoacyl-tRNA biosynthesis | | 2/109 | | 24/829 | 0.84678 | | 0.913088 | | cysS1(1.20869537818),pheS(1.04448125483) | |  |
| ko03030 | DNA replication | | 1/109 | | 16/829 | 0.897482 | | 0.925892 | | rnhB(1.10225217242) | |  |
| ko00052 | Galactose metabolism | | 1/109 | | 18/829 | 0.923143 | | 0.925892 | | bgaB(1.28122412431) | |  |
| ko00550 | Peptidoglycan biosynthesis | | 1/109 | | 18/829 | 0.923143 | | 0.925892 | | ftsI(1.56708045579) | |  |
| ko00051 | Fructose and mannose metabolism | | 1/109 | | 20/829 | 0.942425 | | 0.925892 | | fruK(1.01482926508) | |  |
| ko02024 | Quorum sensing | | 2/109 | | 60/829 | 0.998356 | | 0.939912 | | lepB(1.86960577851),CMM_2975(2.09276850643) | |  |
| ko01230 | Biosynthesis of amino acids | | 5/109 | | 104/829 | 0.999216 | | 0.939912 | | ilvB(1.45377573342),ilvH(1.38644151519),ilvC(1.7277970941),proB(1.08361000488),hisF(1.01287256854) | |  |
| **Δ*rel*-36_vs_WT-36. down KEGG enrichment** | | | | | | | | | | | | |
| ko00190 | Oxidative phosphorylation | | 12/106 | | 24/829 | 7.60E-06 | | 0.000576 | | ctaC(-1.28353164663),ctaD(-1.41118285929),cydB(-1.23731775318),cydA(-1.52101884794),CMM_0970(-1.66032086286),sdhD(-2.12242978614),sdhC(-2.35430438003),sdhB(-1.61744251486),qcrA(-1.29136831202),qcrB(-1.83868208175),ctaE(-1.31260964599),qcrC(-1.1059343576) | |  |
| ko01200 | Carbon metabolism | | 20/106 | | 73/829 | 0.000332 | | 0.01259 | | sucA(-1.32820690554),fbaA(-1.50085552655),icdA(-1.10612878991),folD(-1.23852842948),glpX(-2.06150526611),serC(-1.20082382902),enoA(-1.77645707446),gndA2(-1.28895847276),cysK(-1.08495641773),acnA(-1.64905316715),gapA(-1.03928382314),talA(-1.26256397458),tktA(-1.55025652579),pgiA(-1.06514344716),CMM_0970(-1.66032086286),sdhD(-2.12242978614),sdhC(-2.35430438003),sdhB(-1.61744251486),ackA(-1.07977907398),glyA(-2.0251275891) | |  |
| ko00720 | Carbon fixation pathways in prokaryotes | | 8/106 | | 17/829 | 0.000501 | | 0.012646 | | icdA(-1.10612878991),folD(-1.23852842948),acnA(-1.64905316715),CMM_0970(-1.66032086286),sdhD(-2.12242978614),sdhC(-2.35430438003),sdhB(-1.61744251486),ackA(-1.07977907398) | |  |
| ko00051 | Fructose and mannose metabolism | | 7/106 | | 20/829 | 0.008611 | | 0.163161 | | CMM_1754(-1.4531938013),fbaA(-1.50085552655),glpX(-2.06150526611),gmdA(-2.99451192704),mtlA(-1.04276299708),manB(-1.2948869035),xylA(-1.11863448442) | |  |
| ko00680 | Methane metabolism | | 6/106 | | 17/829 | 0.014368 | | 0.19221 | | fbaA(-1.50085552655),glpX(-2.06150526611),serC(-1.20082382902),enoA(-1.77645707446),ackA(-1.07977907398),glyA(-2.0251275891) | |  |
| ko00020 | Citrate cycle (TCA cycle) | | 7/106 | | 22/829 | 0.015217 | | 0.19221 | | sucA(-1.32820690554),icdA(-1.10612878991),acnA(-1.64905316715),CMM_0970(-1.66032086286),sdhD(-2.12242978614),sdhC(-2.35430438003),sdhB(-1.61744251486) | |  |
| ko00240 | Pyrimidine metabolism | | 10/106 | | 39/829 | 0.019384 | | 0.209871 | | trxB1(-2.44975356934),nrdB(-1.33067102155),nrdA(-1.66936254733),CMM_2224(-1.3024063252),CMM_0900(-1.87984866308),CMM_1126(-1.92211182135),punA(-1.241791813),pyrG(-1.44328938267),pyrB(-1.74313463896),pyrR(-1.16841556678) | |  |
| ko02020 | Two-component system | | 6/106 | | 19/829 | 0.025397 | | 0.240601 | | glnA2(-1.47682018827),glnA1(-3.16248513795),cydB(-1.23731775318),cydA(-1.52101884794),kdpD(-1.31925196503),kdpE(-2.76284422599) | |  |
| ko01220 | Degradation of aromatic compounds | | 3/106 | | 6/829 | 0.030433 | | 0.256278 | | CMM_1706(-2.72420039009),CMM_0541(-1.28842689753),CMM_1729(-1.05462562869) | |  |
| ko00650 | Butanoate metabolism | | 4/106 | | 11/829 | 0.040831 | | 0.259818 | | CMM_0970(-1.66032086286),sdhD(-2.12242978614),sdhC(-2.35430438003),sdhB(-1.61744251486) | |  |
| ko00250 | Alanine, aspartate and glutamate metabolism | | 6/106 | | 21/829 | 0.040997 | | 0.259818 | | CMM_1146(-1.60770462139),glnA2(-1.47682018827),glnA1(-3.16248513795),aldA(-3.18812553504),putA(-1.23471238851),pyrB(-1.74313463896) | |  |
| ko04724 | Glutamatergic synapse | | 2/106 | | 3/829 | 0.044566 | | 0.259818 | | glnA2(-1.47682018827),glnA1(-3.16248513795) | |  |
| ko04727 | GABAergic synapse | | 2/106 | | 3/829 | 0.044566 | | 0.259818 | | glnA2(-1.47682018827),glnA1(-3.16248513795) | |  |
| ko00710 | Carbon fixation in photosynthetic organisms | | 4/106 | | 12/829 | 0.055358 | | 0.279703 | | fbaA(-1.50085552655),glpX(-2.06150526611),gapA(-1.03928382314),tktA(-1.55025652579) | |  |
| ko03420 | Nucleotide excision repair | | 4/106 | | 12/829 | 0.055358 | | 0.279703 | | uvrD2(-1.15266690943),uvrC(-1.12300095905),uvrB(-1.70001228157),uvrA(-2.43748339034) | |  |
| ko00030 | Pentose phosphate pathway | | 6/106 | | 23/829 | 0.061625 | | 0.291907 | | fbaA(-1.50085552655),glpX(-2.06150526611),gndA2(-1.28895847276),talA(-1.26256397458),tktA(-1.55025652579),pgiA(-1.06514344716) | |  |
| ko00750 | Vitamin B6 metabolism | | 3/106 | | 8/829 | 0.070332 | | 0.312607 | | CMM_1526(-2.60156495277),serC(-1.20082382902),CMM_2909(-1.382626364) | |  |
| ko00430 | Taurine and hypotaurine metabolism | | 2/106 | | 4/829 | 0.081739 | | 0.312607 | | aldA(-3.18812553504),ackA(-1.07977907398) | |  |
| ko04066 | HIF-1 signaling pathway | | 2/106 | | 4/829 | 0.081739 | | 0.312607 | | enoA(-1.77645707446),gapA(-1.03928382314) | |  |
| ko03018 | RNA degradation | | 4/106 | | 14/829 | 0.09159 | | 0.312607 | | recQ2(-2.02549168183),enoA(-1.77645707446),CMM_1225(-1.38646680442),dnaK(-1.18851260309) | |  |
| ko00500 | Starch and sucrose metabolism | | 7/106 | | 32/829 | 0.101644 | | 0.312607 | | treZ(-1.168864493),glgC(-1.59548303979),CMM_1398(-1.85108753197),glgB(-2.34778213262),pgiA(-1.06514344716),CMM_1714(-2.70437261819),bglA(-1.03780782635) | |  |
| ko00480 | Glutathione metabolism | | 3/106 | | 10/829 | 0.124734 | | 0.312607 | | icdA(-1.10612878991),CMM_1824(-1.326640512),gndA2(-1.28895847276) | |  |
| ko00362 | Benzoate degradation | | 2/106 | | 5/829 | 0.125054 | | 0.312607 | | CMM_1706(-2.72420039009),CMM_0541(-1.28842689753) | |  |
| ko00910 | Nitrogen metabolism | | 2/106 | | 5/829 | 0.125054 | | 0.312607 | | glnA2(-1.47682018827),glnA1(-3.16248513795) | |  |
| ko00590 | Arachidonic acid metabolism | | 1/106 | | 1/829 | 0.127865 | | 0.312607 | | CMM_1824(-1.326640512) | |  |
| ko00622 | Xylene degradation | | 1/106 | | 1/829 | 0.127865 | | 0.312607 | | CMM_1706(-2.72420039009) | |  |
| ko01051 | Biosynthesis of ansamycins | | 1/106 | | 1/829 | 0.127865 | | 0.312607 | | tktA(-1.55025652579) | |  |
| ko04013 | MAPK signaling pathway - fly | | 1/106 | | 1/829 | 0.127865 | | 0.312607 | | sodA(-2.16173747903) | |  |
| ko04141 | Protein processing in endoplasmic reticulum | | 1/106 | | 1/829 | 0.127865 | | 0.312607 | | hsp20(-1.36440171589) | |  |
| ko04626 | Plant-pathogen interaction | | 1/106 | | 1/829 | 0.127865 | | 0.312607 | | glpK(-1.62888990646) | |  |
| ko04918 | Thyroid hormone synthesis | | 1/106 | | 1/829 | 0.127865 | | 0.312607 | | CMM_1824(-1.326640512) | |  |
| ko00460 | Cyanoamino acid metabolism | | 3/106 | | 11/829 | 0.156197 | | 0.369941 | | CMM_1146(-1.60770462139),bglA(-1.03780782635),glyA(-2.0251275891) | |  |
| ko04212 | Longevity regulating pathway - worm | | 2/106 | | 6/829 | 0.172364 | | 0.395861 | | sodA(-2.16173747903),dnaK(-1.18851260309) | |  |
| ko00630 | Glyoxylate and dicarboxylate metabolism | | 4/106 | | 18/829 | 0.188622 | | 0.408581 | | glnA2(-1.47682018827),glnA1(-3.16248513795),acnA(-1.64905316715),glyA(-2.0251275891) | |  |
| ko00760 | Nicotinate and nicotinamide metabolism | | 3/106 | | 12/829 | 0.18981 | | 0.408581 | | CMM_1126(-1.92211182135),CMM_2028(-1.21107723311),punA(-1.241791813) | |  |
| ko00010 | Glycolysis / Gluconeogenesis | | 6/106 | | 31/829 | 0.194076 | | 0.408581 | | CMM_2516(-1.81874389378),fbaA(-1.50085552655),glpX(-2.06150526611),enoA(-1.77645707446),gapA(-1.03928382314),pgiA(-1.06514344716) | |  |
| ko00361 | Chlorocyclohexane and chlorobenzene degradation | | 1/106 | | 2/829 | 0.239515 | | 0.453818 | | CMM_1706(-2.72420039009) | |  |
| ko00643 | Styrene degradation | | 1/106 | | 2/829 | 0.239515 | | 0.453818 | | CMM_1706(-2.72420039009) | |  |
| ko00791 | Atrazine degradation | | 1/106 | | 2/829 | 0.239515 | | 0.453818 | | CMM_2824(-1.09777953522) | |  |
| ko04211 | Longevity regulating pathway - mammal | | 1/106 | | 2/829 | 0.239515 | | 0.453818 | | sodA(-2.16173747903) | |  |
| ko03440 | Homologous recombination | | 4/106 | | 20/829 | 0.245974 | | 0.454689 | | CMM_2224(-1.3024063252),recR(-1.81504566785),CMM_0900(-1.87984866308),recA(-2.29940672189) | |  |
| ko00220 | Arginine biosynthesis | | 3/106 | | 15/829 | 0.298563 | | 0.520776 | | glnA2(-1.47682018827),glnA1(-3.16248513795),CMM_2824(-1.09777953522) | |  |
| ko03430 | Mismatch repair | | 3/106 | | 15/829 | 0.298563 | | 0.520776 | | CMM_2224(-1.3024063252),uvrD2(-1.15266690943),CMM_0900(-1.87984866308) | |  |
| ko00670 | One carbon pool by folate | | 2/106 | | 9/829 | 0.322953 | | 0.520776 | | folD(-1.23852842948),glyA(-2.0251275891) | |  |
| ko02060 | Phosphotransferase system (PTS) | | 2/106 | | 9/829 | 0.322953 | | 0.520776 | | CMM_1754(-1.4531938013),mtlA(-1.04276299708) | |  |
| ko04112 | Cell cycle - Caulobacter | | 2/106 | | 9/829 | 0.322953 | | 0.520776 | | ftsZ(-2.18447791732),ftsW2(-1.39744548903) | |  |
| ko04146 | Peroxisome | | 2/106 | | 9/829 | 0.322953 | | 0.520776 | | icdA(-1.10612878991),sodA(-2.16173747903) | |  |
| ko04068 | FoxO signaling pathway | | 1/106 | | 3/829 | 0.33699 | | 0.52123 | | sodA(-2.16173747903) | |  |
| ko04213 | Longevity regulating pathway - multiple species | | 1/106 | | 3/829 | 0.33699 | | 0.52123 | | sodA(-2.16173747903) | |  |
| ko00520 | Amino sugar and nucleotide sugar metabolism | | 5/106 | | 31/829 | 0.362607 | | 0.549636 | | galE2(-1.13489310677),gmdA(-2.99451192704),glgC(-1.59548303979),pgiA(-1.06514344716),manB(-1.2948869035) | |  |
| ko00564 | Glycerophospholipid metabolism | | 2/106 | | 10/829 | 0.372508 | | 0.553572 | | glpD(-3.27378473922),pgsA(-1.25673648369) | |  |
| ko00920 | Sulfur metabolism | | 2/106 | | 11/829 | 0.420577 | | 0.592383 | | cysK(-1.08495641773),sseA(-1.0321032559) | |  |
| ko00471 | D-Glutamine and D-glutamate metabolism | | 1/106 | | 4/829 | 0.422073 | | 0.592383 | | murC(-1.0967608706) | |  |
| ko00780 | Biotin metabolism | | 1/106 | | 4/829 | 0.422073 | | 0.592383 | | fabB(-2.07852739734) | |  |
| ko00230 | Purine metabolism | | 7/106 | | 49/829 | 0.439693 | | 0.605893 | | nrdB(-1.33067102155),nrdA(-1.66936254733),CMM_2224(-1.3024063252),CMM_0900(-1.87984866308),relA(-19.8687622805),CMM_1126(-1.92211182135),punA(-1.241791813) | |  |
| ko01230 | Biosynthesis of amino acids | | 14/106 | | 104/829 | 0.463075 | | 0.616663 | | fbaA(-1.50085552655),glnA2(-1.47682018827),icdA(-1.10612878991),glnA1(-3.16248513795),serC(-1.20082382902),enoA(-1.77645707446),dapX(-1.73504135908),cysK(-1.08495641773),CMM_2873(-1.50348064185),acnA(-1.64905316715),gapA(-1.03928382314),talA(-1.26256397458),tktA(-1.55025652579),glyA(-2.0251275891) | |  |
| ko00261 | Monobactam biosynthesis | | 1/106 | | 5/829 | 0.496328 | | 0.616663 | | dapX(-1.73504135908) | |  |
| ko00310 | Lysine degradation | | 1/106 | | 5/829 | 0.496328 | | 0.616663 | | sucA(-1.32820690554) | |  |
| ko00360 | Phenylalanine metabolism | | 1/106 | | 5/829 | 0.496328 | | 0.616663 | | CMM_1729(-1.05462562869) | |  |
| ko00380 | Tryptophan metabolism | | 1/106 | | 5/829 | 0.496328 | | 0.616663 | | sucA(-1.32820690554) | |  |
| ko00473 | D-Alanine metabolism | | 1/106 | | 5/829 | 0.496328 | | 0.616663 | | ddlB(-2.96366101973) | |  |
| ko01210 | 2-Oxocarboxylic acid metabolism | | 3/106 | | 22/829 | 0.549253 | | 0.671413 | | icdA(-1.10612878991),CMM_2873(-1.50348064185),acnA(-1.64905316715) | |  |
| ko00450 | Selenocompound metabolism | | 1/106 | | 6/829 | 0.561121 | | 0.675033 | | trxB1(-2.44975356934) | |  |
| ko00270 | Cysteine and methionine metabolism | | 3/106 | | 23/829 | 0.581003 | | 0.688029 | | cysK(-1.08495641773),sseA(-1.0321032559),CMM_0574(-2.66339263046) | |  |
| ko03320 | PPAR signaling pathway | | 1/106 | | 7/829 | 0.617647 | | 0.720172 | | glpK(-1.62888990646) | |  |
| ko03030 | DNA replication | | 2/106 | | 16/829 | 0.627694 | | 0.720797 | | CMM_2224(-1.3024063252),CMM_0900(-1.87984866308) | |  |
| ko00561 | Glycerolipid metabolism | | 1/106 | | 8/829 | 0.666953 | | 0.729846 | | glpK(-1.62888990646) | |  |
| ko00940 | Phenylpropanoid biosynthesis | | 1/106 | | 8/829 | 0.666953 | | 0.729846 | | bglA(-1.03780782635) | |  |
| ko04122 | Sulfur relay system | | 1/106 | | 8/829 | 0.666953 | | 0.729846 | | sseA(-1.0321032559) | |  |
| ko00052 | Galactose metabolism | | 2/106 | | 18/829 | 0.693044 | | 0.729846 | | CMM_2516(-1.81874389378),galE2(-1.13489310677) | |  |
| ko00550 | Peptidoglycan biosynthesis | | 2/106 | | 18/829 | 0.693044 | | 0.729846 | | murC(-1.0967608706),ddlB(-2.96366101973) | |  |
| ko00260 | Glycine, serine and threonine metabolism | | 3/106 | | 27/829 | 0.693354 | | 0.729846 | | serC(-1.20082382902),CMM_2873(-1.50348064185),glyA(-2.0251275891) | |  |
| ko00130 | Ubiquinone and other terpenoid-quinone biosynthesis | | 1/106 | | 9/829 | 0.709953 | | 0.737082 | | CMM_2831(-1.23039255297) | |  |
| ko00040 | Pentose and glucuronate interconversions | | 1/106 | | 10/829 | 0.747447 | | 0.745376 | | xylA(-1.11863448442) | |  |
| ko00061 | Fatty acid biosynthesis | | 1/106 | | 10/829 | 0.747447 | | 0.745376 | | fabB(-2.07852739734) | |  |
| ko00330 | Arginine and proline metabolism | | 1/106 | | 10/829 | 0.747447 | | 0.745376 | | putA(-1.23471238851) | |  |
| ko00280 | Valine, leucine and isoleucine degradation | | 1/106 | | 11/829 | 0.780134 | | 0.767869 | | CMM_0268(-2.10145588991) | |  |
| ko02024 | Quorum sensing | | 6/106 | | 60/829 | 0.805603 | | 0.775763 | | livM(-2.11909467077),livK(-3.77368075815),CMM_2185(-1.31674757012),kdpE(-2.76284422599),CMM_1588(-1.64385618977),CMM_1587(-2.95899610466) | |  |
| ko01212 | Fatty acid metabolism | | 1/106 | | 12/829 | 0.808625 | | 0.775763 | | fabB(-2.07852739734) | |  |
| ko02010 | ABC transporters | | 10/106 | | 100/829 | 0.854107 | | 0.790326 | | ftsX(-1.67733165004),livM(-2.11909467077),livK(-3.77368075815),CMM_0866(-1.57697968442),CMM_2844(-1.04668768979),CMM_2842(-1.46290421332),CMM_2485(-2.74261142561),gluB(-1.51112317016),gluA(-2.33648028062),gluC(-1.73135483651) | |  |
| ko00300 | Lysine biosynthesis | | 1/106 | | 14/829 | 0.855089 | | 0.790326 | | dapX(-1.73504135908) | |  |
| ko03070 | Bacterial secretion system | | 1/106 | | 14/829 | 0.855089 | | 0.790326 | | tatA(-1.34654467701) | |  |
| ko00640 | Propanoate metabolism | | 1/106 | | 17/829 | 0.904649 | | 0.816224 | | ackA(-1.07977907398) | |  |
| ko03060 | Protein export | | 1/106 | | 17/829 | 0.904649 | | 0.816224 | | tatA(-1.34654467701) | |  |
| ko00620 | Pyruvate metabolism | | 1/106 | | 21/829 | 0.94557 | | 0.843109 | | ackA(-1.07977907398) | |  |
