# Supplementary material for: Bacterial alarmone (p)ppGpp mediates the pathogenicity of Clavibacter michiganensis via a dual mechanism that affects both enzyme production and the Tat secretion system
Source: mSystems. 2025 Aug 4;10(9):e00135-25. doi: 10.1128/msystems.00135-25 (PMC12455917; doi:10.1128/msystems.00135-25)
Supplement: Table S5 — TPM database used in heat map analysis of EPS. [file msystems.00135-25-s0006.docx]

Table S5. TPM (Transcripts Per Million) database used in Heat map analysis (Figure 2A).

|  | **GeneID** | **WT-0_1** | **WT-0_2** | **WT-0_3** | **Δ*rel*-0_1** | **Δ*rel*-0_2** | **Δ*rel*-0_3** | **WT-36_1** | **WT-36_2** | **WT-36_3** | **Δ*rel*-36_1** | **Δ*rel*-36_2** | **Δ*rel*-36_3** |
| --- | --- | --- | --- | --- | --- | --- | --- | --- | --- | --- | --- | --- | --- |
| **EPS I** | CMM_0711 | 198.78 | 234.44 | 265.77 | 211.11 | 220.18 | 208.9 | 311.94 | 382.71 | 321.05 | 469.18 | 528.56 | 514.62 |
|  | CMM_0712 | 213.14 | 187.64 | 197.13 | 164.37 | 165.84 | 165.6 | 199.14 | 176.16 | 178.96 | 216.16 | 214.6 | 204.15 |
|  | CMM_0713 | 37.13 | 38.86 | 39.75 | 34.53 | 31.65 | 37.11 | 23.73 | 24.09 | 36.67 | 44.21 | 49.6 | 40.37 |
|  | CMM_0714 | 143.57 | 137.89 | 129.26 | 116.95 | 104.29 | 98.81 | 19.43 | 26.13 | 13.38 | 19.24 | 25.51 | 21.21 |
|  | *wzy*2 | 167.12 | 167.19 | 166.43 | 142.16 | 136.73 | 134.19 | 83.26 | 72.36 | 84.5 | 48.13 | 50.58 | 47.93 |
|  | *wcnG* | 106.31 | 109.75 | 109.49 | 103.09 | 89.03 | 74.74 | 35.06 | 53.97 | 38.13 | 51.36 | 51.15 | 62.21 |
|  | *wzx2* | 78.35 | 69.33 | 63.82 | 66.73 | 53.38 | 54.48 | 7.16 | 15.36 | 5.54 | 12.7 | 13.92 | 12.57 |
|  | *wcnE* | 82.73 | 95.14 | 95.93 | 66.55 | 63.92 | 59.23 | 19.99 | 35.52 | 23.11 | 38.35 | 38.47 | 37.26 |
|  | *wcnD* | 150.69 | 135.92 | 132.74 | 110.69 | 86.54 | 103.23 | 47.29 | 87.68 | 69.64 | 103.42 | 107.96 | 98.53 |
|  | *wcnC* | 160.79 | 167.56 | 157.78 | 126.59 | 113.63 | 108.8 | 67.74 | 77.18 | 74.82 | 168.35 | 169.72 | 161.14 |
|  | *wzb* | 286.1 | 248.3 | 262.91 | 214.26 | 207.39 | 208.94 | 184.09 | 262.34 | 229.77 | 342.7 | 353.75 | 348.82 |
|  | *wzc* | 975.12 | 977 | 928.83 | 665.8 | 664.24 | 648.83 | 539.78 | 475.8 | 458.98 | 679.14 | 669.94 | 613.7 |
|  | *wcnB* | 410.26 | 431.51 | 414.64 | 332.25 | 318.84 | 326.15 | 152.88 | 128.75 | 151.11 | 45.81 | 46.78 | 45.15 |
|  | *wcnA* | 102.11 | 93.45 | 97.98 | 89.09 | 83.41 | 78.73 | 438.71 | 398.06 | 400.57 | 513.73 | 503.89 | 494.11 |
|  | CMM_0725 | 313.99 | 303.03 | 292 | 242.86 | 239.43 | 265.16 | 591.42 | 488.15 | 630.59 | 423.97 | 458.56 | 434.69 |
|  | CMM_0726 | 457.76 | 422.79 | 394.25 | 399.02 | 399.34 | 410.14 | 150.96 | 196.76 | 161.33 | 298.92 | 323.3 | 333.08 |
|  | CMM_0727 | 546.57 | 578.33 | 546.31 | 502.57 | 419.3 | 438.26 | 50.13 | 69.75 | 59.65 | 36.19 | 35.49 | 43.44 |
| **EPS II** | *wcoA* | 40.74 | 40.18 | 41.32 | 27.5 | 25.95 | 25.21 | 34.48 | 48.23 | 34.27 | 20.66 | 24.27 | 23.27 |
|  | *wcoB* | 0.27 | 0.54 | 0.73 | 0.56 | 0.64 | 1.77 | 5.96 | 9.08 | 7.44 | 6.41 | 7.5 | 6.63 |
|  | *wcoC* | 0.92 | 0.78 | 0.55 | 1.9 | 2.18 | 1.99 | 10.09 | 15.76 | 9.74 | 18.19 | 18.3 | 24.22 |
|  | *wcoF* | 0.14 | 0.65 | 1.19 | 1.18 | 0.67 | 0.92 | 0.31 | 2.38 | 2.13 | 4.22 | 3.38 | 4.02 |
|  | *wcoG* | 3.17 | 3.17 | 3.43 | 3.4 | 3.48 | 2.99 | 1.71 | 2.71 | 2.17 | 2.9 | 3.24 | 2.44 |
|  | *wcoH* | 38.4 | 29.97 | 35.48 | 20.99 | 20.75 | 22.62 | 46.36 | 51.23 | 54.24 | 55.61 | 46.37 | 59.33 |
|  | *wcoI* | 71.74 | 72.58 | 66.55 | 52.69 | 49.08 | 54.02 | 40.18 | 44.2 | 42.13 | 32.54 | 31.87 | 35.92 |
|  | *wcoK* | 7.61 | 11.13 | 7.8 | 9.02 | 6.78 | 11.52 | 122.01 | 152.12 | 124.26 | 128.72 | 157.68 | 180.84 |
|  | CMM_0830 | 37.85 | 42.78 | 40.92 | 37.45 | 30.91 | 35.91 | 229.33 | 336.47 | 242.39 | 315.08 | 330.91 | 372.43 |
|  | *wcoM* | 41.42 | 32.45 | 35.9 | 34.23 | 29.08 | 36.46 | 85.63 | 107.37 | 91.35 | 179.39 | 191.22 | 208.81 |
|  | *wzx*3 | 2.17 | 0.49 | 0.91 | 2.98 | 1.55 | 2.72 | 11.96 | 10.57 | 10.05 | 37.84 | 40.02 | 42 |
|  | *wcoN* | 9.61 | 9.41 | 6.25 | 7.84 | 7.41 | 8.07 | 11.14 | 19.22 | 18.54 | 30.84 | 31.75 | 28.1 |
|  | *wcoO* | 37.05 | 32.92 | 39.7 | 45.85 | 36.89 | 44.01 | 144.74 | 182.39 | 164.33 | 220.65 | 217.2 | 233.98 |
|  | *wcoP* | 69.1 | 62.06 | 73.51 | 65.69 | 58.16 | 77.01 | 301.18 | 363.24 | 313.29 | 421.63 | 424.99 | 408.88 |
|  | *wcoQ* | 43.96 | 46.37 | 40.19 | 31.89 | 34.99 | 41.28 | 107.4 | 149.69 | 107.55 | 212.49 | 218.58 | 241.73 |
|  | *wcoR* | 127.35 | 131.98 | 133.95 | 155.39 | 160.72 | 156.27 | 435.9 | 590.91 | 493.43 | 840.06 | 884.35 | 916.29 |
| **EPS III** | CMM_1005 | 188.32 | 197.6 | 192 | 181.58 | 164.67 | 149.01 | 215.02 | 291.2 | 215.43 | 316.26 | 327.6 | 322.68 |
|  | *wcqB* | 175.91 | 188.07 | 171.83 | 132.03 | 136.77 | 138.9 | 432.36 | 511.06 | 460.78 | 643.49 | 662.34 | 685.08 |
|  | *wzt* | 75.66 | 72.98 | 63.42 | 97.39 | 101.91 | 90.7 | 53.96 | 56.89 | 44.76 | 117.38 | 127.51 | 120.03 |
|  | *wzm* | 3.84 | 0.92 | 2.9 | 2.85 | 2.64 | 3.52 | 4.01 | 2.71 | 1.52 | 4.46 | 3.86 | 3.65 |
|  | *rmlB* | 634.35 | 641.15 | 635.94 | 933.02 | 933.48 | 914.45 | 243.89 | 231.34 | 222.22 | 424.21 | 423.42 | 407.95 |
|  | *wcqC* | 8.55 | 6.19 | 10.23 | 10.64 | 6 | 8.45 | 17.11 | 22.15 | 16.51 | 28.69 | 28.25 | 36.72 |
|  | *rmlC* | 445.67 | 484.19 | 451.35 | 447.65 | 418.26 | 414.38 | 477.09 | 476.3 | 460.32 | 631.24 | 685.84 | 676.18 |
|  | *rmlA* | 393.28 | 370.22 | 391.64 | 333.08 | 359.44 | 396.75 | 173.14 | 224.77 | 194.02 | 295.69 | 313.49 | 306.61 |
|  | *wcqD* | 199.82 | 181.57 | 191.19 | 195.38 | 173.63 | 192.38 | 42.8 | 86.31 | 49.49 | 64.86 | 56.85 | 66.33 |
|  | *wcqE* | 299.75 | 278.88 | 281.36 | 310.71 | 285.12 | 307.5 | 51.82 | 48.62 | 57.98 | 50.56 | 42.69 | 52.16 |
|  | *wcqF* | 428.31 | 428.27 | 407.09 | 420.82 | 416.55 | 428.91 | 214.4 | 172.59 | 196.58 | 146.79 | 137.91 | 143.42 |
|  | *wcqG* | 253.04 | 254.83 | 243.75 | 285.97 | 287.34 | 286.06 | 56.54 | 70.65 | 60.81 | 83.5 | 89.83 | 88.03 |
|  | *wcqH* | 174.96 | 181.69 | 167.05 | 200.39 | 188.72 | 177.48 | 36.62 | 56.39 | 39.75 | 45.93 | 43.35 | 46.01 |
|  | *wcqI* | 87.93 | 104.34 | 83.58 | 93.95 | 88.43 | 104.15 | 159.77 | 193.97 | 161.69 | 224.37 | 200.17 | 213.36 |
|  | *glfA* | 1197.6 | 1160.3 | 1164.43 | 1223.1 | 1286.9 | 1218.9 | 331.29 | 210.95 | 310.34 | 170.48 | 154.83 | 140.94 |
|  | *wcqJ* | 907.86 | 928.35 | 889.08 | 889.74 | 854.85 | 860.67 | 88.3 | 87.41 | 100.46 | 82.92 | 83.75 | 78.71 |
|  | *wcqK* | 134.31 | 134.16 | 137.04 | 117.43 | 107.08 | 106.26 | 65.44 | 63.86 | 59.39 | 44.84 | 45.51 | 45.77 |
|  | *wcqL* | 201.48 | 195.74 | 185.97 | 204.7 | 207.35 | 197.09 | 222.17 | 166.68 | 186.83 | 149.09 | 160.8 | 162.44 |
|  | *wcqM* | 57.73 | 51 | 51.46 | 38.3 | 37.52 | 42.78 | 182.54 | 243.78 | 209.96 | 377.16 | 440.7 | 460.05 |
|  | *wzy4* | 189.94 | 195.5 | 179.41 | 140.14 | 131.72 | 144.41 | 294.92 | 452.61 | 334.82 | 581.82 | 588.48 | 637.44 |
|  | *gcdH* | 61.52 | 55.76 | 52.86 | 88.72 | 88.87 | 91.72 | 154.83 | 198.76 | 158.61 | 628.5 | 653.61 | 651.12 |
|  | *manA* | 236.52 | 239.95 | 239.22 | 211.16 | 192.15 | 176.59 | 181.64 | 193.1 | 199.07 | 134.27 | 136.95 | 139.28 |
|  | *galE*1 | 492.02 | 509.64 | 526.73 | 396.64 | 390.63 | 350.8 | 317.96 | 374.63 | 282.43 | 198.18 | 211.75 | 205.66 |
|  | *whiB1* | 1135.5 | 1124.4 | 1067.38 | 883.85 | 818.76 | 903.42 | 2909.11 | 2975.46 | 2958.17 | 2764.53 | 2938.56 | 3106.83 |
|  | *wcqR* | 246.07 | 236.82 | 227.39 | 196.82 | 175.5 | 162.05 | 400.65 | 509.34 | 427.12 | 625.05 | 622.73 | 679.78 |
|  | CMM_1031 | 253.17 | 231.82 | 230.92 | 189.32 | 171.9 | 165.49 | 166.91 | 216.82 | 187.14 | 151.31 | 157.63 | 159.77 |
| **EPS IV** | *wcmG* | 78.92 | 85.36 | 86.03 | 57.75 | 57.31 | 55.94 | 168.06 | 153.69 | 136.43 | 104.2 | 97.49 | 98.25 |
|  | *wcmE* | 192.86 | 170.97 | 193.02 | 119.33 | 121.99 | 105.77 | 123.24 | 169.57 | 134.12 | 100.18 | 90.18 | 109.92 |
|  | *wcmF* | 384.12 | 358.05 | 374.67 | 265.85 | 276.35 | 279.04 | 187.63 | 126.02 | 192.76 | 60.35 | 66.94 | 67.44 |
|  | *fclA* | 1233.7 | 1177.8 | 1224.41 | 587.39 | 517.52 | 566.19 | 475.48 | 362.38 | 415.03 | 231.23 | 235.06 | 220.56 |
|  | *wcmN* | 221.03 | 226.53 | 221.72 | 86.47 | 83.73 | 85.07 | 986.71 | 936.28 | 915.1 | 372.85 | 392.14 | 411.13 |
|  | *wcmH* | 492.06 | 600.02 | 650.83 | 347.9 | 332.3 | 346.81 | 4591.65 | 3858.05 | 4384.85 | 1342.64 | 1503.67 | 1532.37 |
|  | *gmdA* | 1716.3 | 1585.7 | 1652.11 | 717.1 | 735.83 | 727.78 | 1385.34 | 820.09 | 1168.29 | 159.35 | 138.87 | 142.88 |
|  | *wcmJ* | 325.22 | 322.69 | 327.99 | 156.23 | 142.55 | 156.84 | 731.64 | 460.99 | 669.86 | 220.09 | 206.66 | 208.71 |
|  | *wcmI* | 285.61 | 335.06 | 285.79 | 147.08 | 124.05 | 125.31 | 1570.76 | 1277.39 | 1419.25 | 392.6 | 391.27 | 436.56 |
|  | *wzy*1 | 182.23 | 185.75 | 184 | 91.7 | 79.32 | 77.96 | 35.92 | 44.59 | 38.04 | 44.27 | 41.99 | 41.92 |
|  | *wcmM* | 215.88 | 200.82 | 187.17 | 131.01 | 122.82 | 160.26 | 43.83 | 56.22 | 71.59 | 76.21 | 92.41 | 81 |
|  | CMM_1606 | 259.86 | 265.09 | 240.6 | 169.07 | 186.26 | 175.06 | 86.3 | 86.57 | 86.82 | 73.97 | 75.53 | 76.61 |
|  | *wzx*1 | 89.82 | 88.33 | 78.17 | 65.71 | 49.9 | 51.17 | 22.3 | 38.52 | 21.92 | 35.67 | 38.13 | 37.14 |
|  | CMM_1610 | 75.19 | 75.16 | 59.22 | 68.48 | 47.93 | 55.82 | 314.89 | 432.45 | 310.13 | 417.6 | 428.35 | 469.89 |
|  | *cspA*1 | 18087 | 15451 | 16974 | 15244 | 17160 | 19390 | 7882.21 | 6984.8 | 8581.94 | 1366.42 | 1454.22 | 1205.19 |
