# Supplementary material for: Bacterial alarmone (p)ppGpp mediates the pathogenicity of Clavibacter michiganensis via a dual mechanism that affects both enzyme production and the Tat secretion system
Source: mSystems. 2025 Aug 4;10(9):e00135-25. doi: 10.1128/msystems.00135-25 (PMC12455917; doi:10.1128/msystems.00135-25)
Supplement: Table S6 — TPM database used in heat map analysis of virulence genes. [file msystems.00135-25-s0007.docx]

Table S6. TPM (Transcripts Per Million) database used in Heat map analysis (Figure 2B).

|  | **Gene ID** | **WT-0_1** | **WT-0_2** | **WT-0_3** | **Δ*rel*-0_1** | **Δ*rel*-0_2** | **Δ*rel*-0_3** | **WT-36_1** | **WT-36_2** | **WT-36_3** | **Δ*rel*-36_1** | **Δ*rel*-36_2** | **Δ*rel*-36_3** |
| --- | --- | --- | --- | --- | --- | --- | --- | --- | --- | --- | --- | --- | --- |
| **Secretory protein** | *chpG* | 33.14 | 22.83 | 32.06 | 43.97 | 43.38 | 60.82 | 302.98 | 432.45 | 260.62 | 123.74 | 428.35 | 116.21 |
|  | CMM_1557 | 2830.5 | 2759.39 | 2695.7 | 2116.53 | 2031.8 | 2035.2 | 3118.34 | 1739 | 2281.17 | 87.54 | 99.25 | 103.71 |
|  | *ppaG* | 76.61 | 77.01 | 85.32 | 115.32 | 85.99 | 106.59 | 210.8 | 253.9 | 212.53 | 231.54 | 236.81 | 247.78 |
|  | *sbtA* | 7.04 | 7.11 | 7.26 | 11.26 | 12.07 | 14.31 | 18.62 | 32.73 | 20.14 | 44.65 | 49.04 | 47.87 |
|  | *sbtB* | 150.71 | 184.69 | 182.53 | 52.03 | 46.7 | 44.25 | 260.9 | 254.8 | 263.68 | 93.54 | 102.59 | 105.03 |
|  | *xysB* | 2.54 | 3.59 | 2.1 | 6.98 | 5.41 | 6.09 | 4.05 | 4.7 | 4.38 | 10.04 | 8.45 | 11.28 |
|  | *xysA* | 17.52 | 20.26 | 18.68 | 36.86 | 44.54 | 36.33 | 8.37 | 15.03 | 8.83 | 30.98 | 28.17 | 32.32 |
|  | CMM_0430 | 28.33 | 31.59 | 24.09 | 16.15 | 12.02 | 14.56 | 36.06 | 38.77 | 31.55 | 8.81 | 10.11 | 11.26 |
|  | CMM_2768 | 451.87 | 450.51 | 427.88 | 481.69 | 458.5 | 451.99 | 712.21 | 522 | 649.94 | 39.02 | 35.27 | 43.2 |
|  | *tatA* | 1461.55 | 1219.37 | 1348.8 | 1115.32 | 1384.5 | 1320.4 | 1504.17 | 1384.1 | 1580.13 | 628.05 | 625.15 | 577.65 |
|  | *tatC* | 651.63 | 638.26 | 652.94 | 632.45 | 586.29 | 618.42 | 461.67 | 507.17 | 523.55 | 428.15 | 384.5 | 385.94 |
| **Biofilm** | CMM_1144 | 116.48 | 130.58 | 135.21 | 208.27 | 203.25 | 190.29 | 211.39 | 132.02 | 205.71 | 44.65 | 44.5 | 49.97 |
|  | CMM_1147 | 105.23 | 92.13 | 87.38 | 78.08 | 74.18 | 77.19 | 283.98 | 369.99 | 295.46 | 413.22 | 427.97 | 459.05 |
|  | CMM_1146 | 35.52 | 25.2 | 34.07 | 53.54 | 43.82 | 43.65 | 442.33 | 277.87 | 388.96 | 118.73 | 121.67 | 138.68 |
|  | CMM_2683 | 2.33 | 2.53 | 2.53 | 3.04 | 2.9 | 3.99 | 23.73 | 35.77 | 21.39 | 69.31 | 63.97 | 71.31 |
|  | *gluA* | 473.73 | 458 | 458.22 | 389.56 | 389.1 | 403.17 | 1116 | 1034.7 | 1197.84 | 260.12 | 215.12 | 215.6 |
|  | *wcmN* | 221.03 | 226.53 | 221.72 | 86.47 | 83.73 | 85.07 | 986.71 | 936.28 | 915.1 | 372.85 | 392.14 | 411.13 |
|  | *trpE2* | 23.09 | 26.83 | 29.77 | 23.95 | 26.86 | 30.46 | 145.07 | 185.06 | 160.19 | 169.07 | 157.07 | 203.47 |
|  | *glgP* | 246.53 | 251.86 | 247.22 | 137.11 | 128.37 | 138.23 | 70.51 | 102.3 | 80.73 | 66.33 | 76.42 | 89.62 |
|  | CMM_1398 | 107.08 | 105.41 | 105.11 | 31.83 | 31.49 | 28.42 | 645.39 | 598.27 | 602.25 | 397 | 415.72 | 408.56 |
|  | *glgB* | 64.29 | 91.74 | 78.62 | 16.47 | 16.26 | 15.3 | 578 | 574.39 | 581.33 | 345.73 | 347.82 | 350.23 |
|  | *cysE* | 160.11 | 209.33 | 223.88 | 236.73 | 246.67 | 243.75 | 329.92 | 414.7 | 394.86 | 308.05 | 337.12 | 333.94 |
|  | *glgC* | 1051.68 | 1088.28 | 1042.6 | 703.72 | 718.65 | 700.56 | 385.06 | 319.85 | 355.32 | 129.95 | 125.51 | 110.18 |
|  | CMM_2525 | 19.7 | 21.02 | 19.63 | 13.96 | 11.21 | 16.73 | 382.59 | 291.69 | 301.02 | 100.61 | 101.97 | 105.74 |
|  | CMM_0054 | 24.53 | 24.33 | 18.02 | 10.61 | 10.2 | 9.71 | 162.35 | 88.1 | 132.65 | 19.49 | 15.87 | 15.8 |
| **Quorum sensing** | CMM_0267 | 14.57 | 17.52 | 13.5 | 13.11 | 9.26 | 12.27 | 869.52 | 372.18 | 634.86 | 78.23 | 52.33 | 61.68 |
|  | CMM_0674 | 63.11 | 65.43 | 65.22 | 62.7 | 64.46 | 56.08 | 74.33 | 60.64 | 82.71 | 18.01 | 17.72 | 20.83 |
|  | CMM_0893 | 90.28 | 87.63 | 87.42 | 28.66 | 26.7 | 30.19 | 317.2 | 315.26 | 277.17 | 72.19 | 68.61 | 77.64 |
|  | CMM_1145 | 146.62 | 137.44 | 144.87 | 244.46 | 284.24 | 260.14 | 651.15 | 215.97 | 460.24 | 19.81 | 19.31 | 12.9 |
|  | CMM_2867 | 317.44 | 285.01 | 287.13 | 279.52 | 291.51 | 273.01 | 1152.6 | 1285.3 | 1205.68 | 149.89 | 145.32 | 148.91 |
|  | *kdpD* | 61.87 | 59.88 | 63 | 44.11 | 42.29 | 44.04 | 93.28 | 100.35 | 75.32 | 35.19 | 38.88 | 38.23 |
|  | CMM_2567 | 32.67 | 35.5 | 43.48 | 54.97 | 48.67 | 52.16 | 186.06 | 254.3 | 200.62 | 477.63 | 447.98 | 474.22 |
|  | *kdpE* | 39.71 | 42.36 | 39.72 | 29.84 | 28.48 | 33.39 | 26.94 | 40.19 | 36.2 | 4.68 | 6.25 | 4.93 |
|  | *lepB* | 277.07 | 260.57 | 229.58 | 217.09 | 223.57 | 255.7 | 323.22 | 414.67 | 373.96 | 1370.75 | 1460.3 | 1402.01 |
|  | *livH* | 305.36 | 310.71 | 297.04 | 286.87 | 258.08 | 285.33 | 501.09 | 274.73 | 395.12 | 216.42 | 197.17 | 211.54 |
|  | *livK* | 1853.15 | 1799.11 | 1807.7 | 2220.84 | 2316.9 | 2354.3 | 5197.3 | 2690.7 | 4081.55 | 336.14 | 285.78 | 290.04 |
| **Transcription factor** | *rsrA* | 295.61 | 289.5 | 286.82 | 190.57 | 198.8 | 192.18 | 1891.53 | 1506 | 1944.64 | 265.83 | 297.01 | 316.72 |
|  | *sigB* | 433.24 | 431.94 | 429.19 | 249.35 | 234.97 | 242.36 | 1183.83 | 929.5 | 1226.83 | 237.83 | 214.43 | 226.08 |
|  | *sigH* | 226.21 | 203.82 | 204.69 | 141.48 | 146.52 | 154.51 | 1928.85 | 1956.1 | 2248.48 | 236.3 | 226.59 | 244.68 |
|  | *sigK* | 326.5 | 298.77 | 319.94 | 226.66 | 227.53 | 218.92 | 4196.88 | 2730.6 | 3728.37 | 668.92 | 729.16 | 674.67 |
|  | *sigL* | 39.93 | 47.44 | 44.44 | 42.64 | 23.1 | 31.71 | 278.25 | 223.53 | 291.59 | 140.42 | 170.08 | 184.31 |
|  | *sigY* | 128.65 | 121.54 | 126.5 | 83.08 | 68.53 | 90.11 | 119.7 | 133.39 | 130.92 | 86.94 | 99.96 | 107.07 |
| **Others** | CMM_0320 | 54.17 | 55.96 | 62.39 | 35.38 | 27.41 | 31.08 | 83.92 | 104.26 | 99.03 | 17.68 | 15.54 | 15.94 |
|  | CMM_1644 | 540.03 | 498.99 | 497.26 | 334.84 | 360.13 | 338.74 | 660.65 | 697.29 | 752.9 | 87.62 | 93.17 | 88.49 |
|  | *manC* | 468.71 | 467.29 | 455.47 | 311.31 | 300.46 | 321.38 | 672.87 | 611.31 | 599.2 | 327.62 | 354.91 | 326.26 |
|  | *npsA* | 0.36 | 0.67 | 0.58 | 0.64 | 0.49 | 1.06 | 19.52 | 31.11 | 20.44 | 12.98 | 13.22 | 16.42 |
|  | pCM1_0001 | 232.23 | 217.6 | 242.65 | 142.61 | 157.35 | 186.84 | 2140.79 | 2006.9 | 2103.57 | 187.51 | 209.8 | 228.97 |
|  | *pknC* | 90.52 | 86.89 | 92.08 | 42.75 | 34.58 | 46.86 | 115.17 | 105.38 | 106.56 | 27.33 | 27.5 | 32.75 |
