# Supplementary material for: Bacterial alarmone (p)ppGpp mediates the pathogenicity of Clavibacter michiganensis via a dual mechanism that affects both enzyme production and the Tat secretion system
Source: mSystems. 2025 Aug 4;10(9):e00135-25. doi: 10.1128/msystems.00135-25 (PMC12455917; doi:10.1128/msystems.00135-25)
Supplement: Table S7 — Strains and plasmids used. [file msystems.00135-25-s0008.docx]

Table S7. Strains and plasmids used in the current study.

| **Strain Name** | **Genotype** | **Reference** |
| --- | --- | --- |
| *Escherichia coli* |  |  |
| *Trans*5*α* | F^-^ φ80d *lac*ZΔM15 Δ(*lac*ZYA-*arg*F) U169 *end* A1 *recA*1 *hsd*R17(r_k_ ^-^ ,m_k_ ^+^ ) *sup*E44λ-*thi*-1 *gyr*A96 *rel*A1 *pho*A | TransGen Biotech |
| BL21 (DE3) | F^-^ *omp*T *hsd*S_B_ (r_B_ ^-^ , m_B_ ^-^ ) *gal* *dcm* (DE3) | TransGen Biotech |
| MC4100 | F^-^ △*lac* U169 araD139 *rps*L150 *rel*A1 ptsFrbs flb5301 | [1] |
| Δ*ssamiAC* | *amiA*Δ2–33, *amiC*Δ2–32, MC4100 derivative | [1] |
| *Clavibacter michiganensis* | | |
| BT0505 | *C. michiganensis* wild-type | Laboratory |
| EV | Control isolate: *C. michiganensis* BT0505 containing pHN216, Neo^R^ | [2] |
| Δ*rel* | *C. michiganensis rel* deletion mutant, BT0505 derivative, Cm^R^ | [2] |
| Δ*rel*(*rel*) | Complementation isolate: Δ*rel* containing pHN216*rel*, Cm^R^ Neo^R^ | [2] |
| OE*vatr1* | *C. michiganensis vatr1* overexpression mutant, BT0505 derivative, containing pHN216-*J23119*-vatr1, Neo^R^ | The current study |
| *ΔxysB* | *C. michiganensis xysB* deletion mutant, BT0505 derivative | The current study |
| *ΔxysB*::*xysB* | Complementation isolate: *ΔxysB*, containing pHN216-xysB-Flag, Neo^R^ | The current study |
| OE*xysB* | *C. michiganensis xysB* overexpression mutant, BT0505 derivative, containing pHN216-xysB-Flag, Neo^R^ | The current study |
| Plasmid |  |  |
| p*EASY*-Blunt Simple | Simple cloning vector, Neo^R^ | TransGen Biotech |
| pHN216 | E. coli-Clavibacter shuttle vector. Gen^R^ , Neo^R^ | [3] |
| pHN216-katA-codAupp | Vector including the *codA*::*upp* cassette for unmarked knockout of *katA* by CRISPR/Cas9-mediated transformation. Neo^R^, 5-FC^S^ | [4] |
| pHN216*xysB*sg1779-codAupp | Derivative of pHN216-katA-codAupp for unmarked knockout of *xysB* by CRISPR/Cas9-mediated transformation. Neo^R^, 5-FC^S^ | The current study |
| pHN216-*J23119*-vatr1 | pHN216 containing the *C. michiganensis* *vatr1* gene and *J23119* promoter; NeoR | The current study |
| pHN216-xysB-Flag | pHN216 containing the *C. michiganensis* *xysB* gene and its native promoter; NeoR | The current study |
| PGEX-GST-Vatr1 | Vectors for fusion expression of Vatr1 and GST protein tags | The current study |
| PGEX-His-Vatr1-GFP | Vectors for fusion expression of 6×His,Vatr1, and GFP protein tags | The current study |
| pssamiA-amiAH | ssAmiA fused to mature *AmiA* carrying a C-terminal hexa histidine tag in pSP72-Ptat | [1] |
| pssxysA-amiAH | As pssAmiA-AmiAH but where AmiA signal peptide has been replaced by *xysA* signal peptide | The current study |
| pssxysB-amiAH | As pssAmiA-AmiAH but where AmiA signal peptide has been replaced by *xysB* signal peptide | The current study |

References:

1. Yan X, Hu S, Yang Y, Xu D, Li H, Liu W, et al. The twin-arginine translocation system is important for stress resistance and virulence of *Brucella melitensis*. Infect Immun. 2020;88(11):10.1128/iai.00389-20. doi: 10.1128/iai.00389-20.

2. Bai K, Jiang N, Chen X, Xu X, Li J, Luo L. RNA-Seq analysis discovers the critical role of Rel in ppGpp synthesis, pathogenicity, and the VBNC state of *Clavibacter michiganensis*. Phytopathology. 2022;112(9):1844-58. doi: 10.1094/phyto-01-22-0023-r. PubMed PMID: 35341314.

3. Laine, MJ., Nakhei, H, Dreier, J, Lehtilä, K, Meletzus, D, Eichenlaub, R, *et al.* Stable transformation of the gram-positive phytopathogenic bacterium *Clavibacter michiganensis* subsp. *sepedonicus* with several cloning vectors. Appl Environ Microbiol. 1996; 62(5):1500-6. doi: 10.1128/aem.62.5.1500-1506.1996.

4. Chen X, Tan Q, Lyu Q, Yu C, Jiang N, Li J, et al. Unmarked gene editing in *Clavibacter michiganensis* using CRISPR/Cas9 and 5-fluorocytosine counterselection. Mol Plant Microbe Interact. 2022;35(1):4-14. doi: 10.1094/mpmi-07-21-0179-ta. PubMed PMID: 34543054.
