# Supplementary material for: Bacterial alarmone (p)ppGpp mediates the pathogenicity of Clavibacter michiganensis via a dual mechanism that affects both enzyme production and the Tat secretion system
Source: mSystems. 2025 Aug 4;10(9):e00135-25. doi: 10.1128/msystems.00135-25 (PMC12455917; doi:10.1128/msystems.00135-25)
Supplement: Table S8 — Primers used for the qPCR analysis. [file msystems.00135-25-s0009.docx]

Table S8. Primers used for the qPCR analysis conducted in the current study.

| **Primer** | **Sequence (5’→3’)** | **Target** | **Refence** |  |  |
| --- | --- | --- | --- | --- | --- |
| **qRT-PCR** | | | | |  |
| gyrBRT-F | GGACAGCACATCACGACCC | *gyrB* | (Jiang et al., 2019) |  |  |
| gyrBRT-R | CCTTCGGCATCTTCTTCCC |  |  |  |  |
| bipART-F | GGGTGCTGGTCGTCGTA | *bipA* | (Jiang et al., 2019) |  |  |
| bipART-R | CGAGCCGCTGTTCAAG |  |  |  |  |
| xysBRT-F | TGGAAGAGGTCGCGGTAGTA | *xysB* | The current study |  |  |
| xysBRT-R | TGAACGCGTCGAACCAGAAC |  |  |  |  |
| xysART-F | ACGGGCAACAGCGAGAAC | *xysA* | The current study |  |  |
| xysART-R | GATCATCGTGAACAGGTCCTTG |  |  |  |  |
| tomART-F | ACTCGGTGGATCCCTTCGTA | *tomA* | The current study |  |  |
| tomART-R | TGAAGTCAGGGATCAACGCC |  |  |  |  |
| celART-F | GGTTCTCCGCATCAAACTATCC | *celA* | The current study |  |  |
| celART-R | TGCTTGTCGCTCGTCGTC |  |  |  |  |
| ppaART-F | AATCGGGCTGGTTCTGGTTT | *ppaA* | The current study |  |  |
| ppaART-R | AGATTCTGCGGCATCTGCAT |  |  |  |  |
| pelART-F | GTGCGTTCCTGCGGTAAC | *pelA* | The current study |  |  |
| pelART-R | GCGGATGGTGATGTGGTC |  |  |  |  |
| tatART-F | TCGTGCTGCTCTTCGGTTCCACG | *tatA* | The current study |  |  |
| tatART-R | GGAGCCGCTCTCCTCCTTCATGG |  |  |  |  |
| tatBRT-F | GTCGAGCTTCTTCCAGTC | *tatB* | The current study |  |  |
| tatBRT-R | ATGCTCATCGGCATCATC |  |  |  |  |
| tatCRT-F | TTCGGCTTCTTCTTCTCCGC | *tatC* | The current study |  |  |
| tatCRT-R | AGCTTCAGCACGAAGTCGAA |  |  |  |  |
| gluART-F | CGCGAAGAGGTTGAAGGACT | *gluA* | The current study |  |  |
| gluART-R | GATCAACCGGCTTGAGACCA |  |  |  |  |
| glgBRT-F | TGTACGAGCACACCGACC | *glgB* | The current study |  |  |
| glgBRT-R | GTGGAACTCCTCGAACCAGT |  |  |  |  |
| glgCRT-F | GATCTTCAGCCAGCAGCTCA | *glgC* | The current study |  |  |
| glgCRT-R | ACCTTGTCGAACACGATGCT |  |  |  |  |
| Cmm_2525RT-F | ATCTGCCTGCTGATGCTCG | Cmm_2525 | The current study |  |  |
| Cmm_2525RT-R | GATCCTCACCAAGACCACGG |  |  |  |  |
| treXRT-R | CTGCTGGCCCCGCTTTCACC | *treX* | The current study |  |  |
| treXRT-F | TCGGGCTGGTTGGCGTCATT |  |  |  |  |
| Cmm_0765RT-F | TGCATCTCCTCGACCCAGTA | Cmm_0765 | The current study |  |  |
| Cmm_0765RT-R | TCGACAACGCGTCCTACTAC |  |  |  |  |
| Cmm_1398RT-F | CAGCTGCTCTTCATGGGTCA | Cmm_1398 | The current study |  |  |
| Cmm_1398RT-R | GACCTCGGTGAGGTACTCCT |  |  |  |  |
| Cmm_1714RT-F | TCGGGTTGTCGAAGTTGAGG | Cmm_1714 | The current study |  |  |
| Cmm_1714RT-R | GACACCGAGGAGTCCAACTG |  |  |  |  |
| **xysB gene editing** | | | | |  |
| xysBupF | GCGGCAGCGTGAAGCTTGTTAACGGCAGACCGTTCCGCTGG | xysB upsteam for pHN216*xysB*sg1779-codAupp | The current study |  |  |
| xysBupR | TTACACTTTATGCTCCCGGCCGTGGTTCTTCCTCGACAACG |  |  |  |  |
| xysBdownF | CGAGAACAGGGGCTGGTGAAGGCAGACCGTTCCGCTGG | xysB downsteam for pHN216*xysB*sg1779-codAupp | The current study |  |  |
| xysBdownR | tgcTTTTTTTCGTGGTTCTTCCTCGACAACGG |  |  |  |  |
| CxysBsgRNAF | AAGAACCACGAAAAAAAgcaccgactcggtg | xysBsgRNA | The current study |  |  |
| CxysBsg1977revR | TTAAGTATTTCACGGCTTAAGttgacagctagctcagtcctaggtataatactagtGGACGTGGATGACATCACGGgttttagagctagaaatagc |  |  |  |  |
| xysBNPF | CTGAGTGCTTGCGGCAGCGTGAAGCTTGATGATCCGGCAGGAGGCCGCGA | *xysB* gene containing native promoter for pHN216-xysB-Flag | The current study |  |  |
| FxysBR | GTGGCGGTACTTGGGTCGATATCTCACTTATCGTCATCGTCCTTGTAATCGATGTCGTGATCCTTATAGTCTCCATCATGGTCTTTGTAGTCCATCGAGCCGGCGCGCGTCACGAT |  |  |  |  |
| oevatr1-F | GGGTACCGCTCTTAAGATGACTCGCGCCGCCCTCG | *vatr1* gene for pHN216-*J23119*-vatr1 | The current study |  |  |
| oevatr1-R | GGTACTTGGGTCGATATCTCAGACGGCGGCGGCCCG |  |  |  |  |
| **Protein purification** | | | | | |
| Vatr1GST-F | CCGCGTGGATCCCCGATGACTCGCGCCGCCCTCGCCA | *vatr1* gene for PGEX-GST-Vatr1 | The current study |  |  |
| Vatr1GST-R | TCGAGTCGACCCGGGTCAGACGGCGGCGGCCCGCTCG |  |  |  |  |
| vatr1-MST-F | CCATCACCATCACCCCCATATGACTCGCGCCGCCCTC | *vatr1* gene for PGEX-His-Vatr1-GFP | The current study |  |  |
| vatr1-MST-R | CTTTACTCATGACGGCGGCGGCCCGCTC |  |  |  |  |
| GFP-MST-F | ACCATCACCCCCATATGAGTAAAGGAGAAGAACTTTTCA | *gfp* gene for PGEX-His-Vatr1-GFP | The current study |  |  |
| GFP-MST-R | GTGGTGGTGGTGGTGCTCGAGTTTGTATAGTTCATCCATGCCATGT |  |  |  |  |
| PGEX-F | TGACCCGGGTCGACTCGAG | linearised PGEX | The current study |  |  |
| PGEX-R | GAATACTGTTTCCTGTGTGAAATTG |  |  |  |  |
| **Amidase reporter assay** | | | | |  |
| pSPPxysAF | TCTACCACAGAGGAACATGTATGACCCTCATCCCACGCCGT | *xysA* signal peptide for pssxysA-amiAH | The current study |  |  |
| pSPPxysAR | CTGGTTTTTAAAAGTTCGTCTTTGGCGGCGGATGCCGAACCCGC |  |  |  |  |
| pSPPxysBF | TCTACCACAGAGGAACATGTATGAGCATTCCCCGCACCAG | *xysA* signal peptide for pssxysB-amiAH | The current study |  |  |
| pSPPxysBR | CTGGTTTTTAAAAGTTCGTCTTTGGCCTGTGCGGACGGCGCGGCGA |  |  |  |  |
| **Probe** | | | | |  |
| xysBProbeF | GAGTCGGTCACCTTCTGGGGCTCC | *xysB* promoter probe for EMSA | The current study |  |  |
| xysBProbeR | CGTCGAACACCTCTCATGTGTTCCGAC |  |  |  |  |
